# Angiotensin-(1-7) in asthma and pulmonary fibrosis: mechanisms of action, clinical safety, and nano-delivery strategies

**DOI:** 10.3389/fphar.2026.1789833

**Published:** 2026-08-03

**Authors:** Youssuf Khanafer, Ayman Dalol, Ahmed Z. El-Hashim, Ibrahim F. Benter, Saghir Akhtar

**Affiliations:** 1 College of Medicine, QU Health, Qatar University, Doha, Qatar; 2 Department of Pharmacology and Therapeutics, Faculty of Pharmacy, Kuwait University, Kuwait, Kuwait; 3 Faculty of Pharmacy, Final International University, Kyrenia, Cyprus

**Keywords:** angiotensin-(1-7), asthma, drug delivery systems, nanotechnology, patient safety, pulmonary fibrosis

## Abstract

**Background/Objectives:**

Asthma and pulmonary fibrosis (PF) are chronic respiratory diseases with distinct etiologies yet convergent pathophysiological mechanisms, including persistent inflammation, aberrant tissue remodeling, and progressive fibrosis. Together, these conditions affect over 260 million individuals worldwide and impose a substantial clinical and socioeconomic burden. Despite advances in pharmacotherapy, current treatments remain largely symptomatic and fail to adequately modify disease progression, underscoring the urgent need for novel therapeutic strategies. Angiotensin-(1–7) (Ang-(1–7)), a bioactive heptapeptide of the protective arm of the renin–angiotensin system, has emerged as a critical regulator of pulmonary homeostasis and injury responses. This review aims to synthesize current evidence supporting the therapeutic potential of Ang-(1–7) in asthma and PF, while assessing translational challenges and delivery solutions.

**Discussion:**

Preclinical studies consistently demonstrate that Ang-(1–7) attenuates airway hyperresponsiveness, suppresses pro-inflammatory cytokine release, reduces oxidative stress, and limits pathological tissue remodeling and fibrosis, collectively resulting in improved lung function and preservation of pulmonary architecture. Clinical trial data from diverse disease settings indicate a favorable safety and tolerability profile for Ang-(1–7), however, disease-specific clinical trials in asthma and PF remain lacking and are necessary for definitive clinical translation. Therapeutic application of Ang-(1-7) is further constrained by rapid proteolytic degradation, poor oral bioavailability, and limited penetration across physiological barriers. Nonetheless, emerging nanotechnology-based delivery platforms show promise in enhancing peptide stability, pharmacokinetics, and lung-targeted delivery.

**Conclusion:**

Taken together, current evidence positions Ang-(1–7)–based formulations as promising disease-modifying candidates for asthma and pulmonary fibrosis. Continued optimization of delivery strategies and well-designed, disease-specific clinical trials are warranted to advance Ang-(1–7) toward clinical application in chronic pulmonary disease.

## 1 Introduction

Asthma and pulmonary fibrosis (PF) are chronic pulmonary diseases with distinct etiologies that have overlapping pathophysiological features, particularly in the domains of inflammation, fibrosis, and remodeling (Huang et al., 2025). Despite therapeutic advances, both conditions continue to pose major clinical challenges not solved by current pharmaceuticals. In asthma, a chronic inflammatory airway disease characterized by airway hyperresponsiveness, mucus hypersecretion, and structural remodeling, approximately 260 million people worldwide were affected in 2021 (Oh et al., 2025). Of these patients, many remain poorly controlled despite the widespread use of inhaled corticosteroids and advent of monoclonal antibodies therapy in asthma (Ishmael et al., 2024). A subset of around 5%–10% of asthmatic patients exhibit steroid resistance, including both Th2 and non-Th2 endotypes, making them particularly difficult to treat (Nabe, 2020). Regarding fibrosis, it is a clinically significant manifestation in the pathogenesis of asthma, often serving as an indicator of severity, therapy resistance, and long-term exacerbations that can be life-threatening (Savin et al., 2023). Airway fibrosis in asthma is a consequence of persistent injury and dysregulated repair mechanisms, thereby promoting extracellular matrix deposition, airway wall thickening, and fixed airflow obstruction (Khalfaoui and Pabelick, 2023).

On the other hand, PF is a chronic interstitial lung disease marked by abnormal wound healing that leads to scarring, impaired gas exchange, progressive breathlessness, and ultimately respiratory failure (Koudstaal et al., 2023). Among these, idiopathic pulmonary fibrosis (IPF) represents the most prominent and devastating manifestation. IPF is a progressive and irreversible interstitial lung disease of unknown cause, mainly characterized by excessive fibrotic tissue accumulation in the lung parenchyma, leading to impaired gaseous exchange and respiratory failure (Wen et al., 2025). It is primarily treated with antifibrotic agents like pirfenidone (a TGF-β pathway modulator) and nintedanib (a tyrosine kinase inhibitor targeting multiple pro-fibrotic receptors), which modestly slow the progression of IPF but do not reverse it, nor do they demonstrate a significant impact on survival. Recent phase III trials have shown disappointing outcomes for novel agents like zinpentraxin alfa (a recombinant human pentraxin-2 that modulates macrophage activity and fibrocyte differentiation), ziritaxestat (an autotaxin inhibitor that blocks lysophosphatidic acid synthesis), and pamrevlumab (a monoclonal antibody against connective tissue growth factor, CTGF), further reinforcing the urgent need for novel therapeutic approaches (Trachalaki et al., 2025). Given that airway fibrosis in asthma and interstitial fibrosis in PF both arise from chronic inflammation and excessive tissue remodeling, their overlapping features suggest that targeting these shared pathways, like those modulated by the renin-angiotensin system (RAS), could offer therapeutic benefits across both conditions.

**FIGURE 1:**
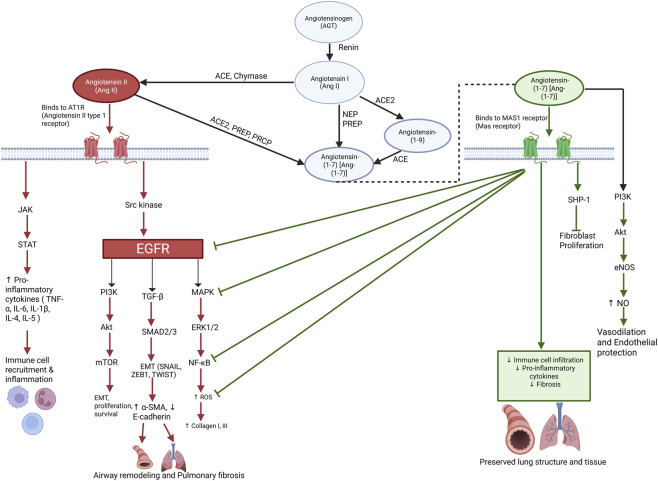
Integrated signaling map of the renin–angiotensin system (RAS) and EGFR crosstalk in asthma and PF. The figure illustrates how Angiotensin II (Ang II), produced via ACE from angiotensin I, binds to AT1R and activates downstream pro-inflammatory and fibrotic pathways via JAK/STAT, PI3K/Akt/mTOR, TGF-β/SMAD2/3, and MAPK/ERK/NF-κB signaling. These cascades promote immune cell recruitment, cytokine release (TNF-α, IL-6, IL-1β, IL-4, IL-5), epithelial–mesenchymal transition (EMT), collagen deposition, and airway remodeling. EGFR acts as a central node amplifying fibrotic signaling. In contrast, Angiotensin-(1–7), generated from Ang II or Ang I via ACE2 (or alternatively via neprilysin (NEP), prolylendopeptidase (PREP), and prolylcarboxypeptidase (PRCP)) binds to the Mas receptor and activates protective pathways including PI3K/Akt/eNOS and SHP-1. These pathways inhibit fibroblast proliferation, suppress immune infiltration and cytokine release, enhance NO production, and preserve lung architecture. The figure highlights the balance between pathogenic Ang II–EGFR-driven responses and the protective Ang-(1–7)–Mas axis in lung inflammation and fibrosis.

The renin-angiotensin-system (RAS) has traditionally been recognized for its crucial role in regulating blood pressure through fluid volume and electrolyte balance modulation, yet this system has recently emerged as an influential regulator of diverse physiological processes (Bernstein et al., 2013; Akhtar et al., 2020). Its relevance in broader pathological contexts is emphasized in accumulating evidence that reveals RAS as a sophisticated molecular network intricately involved in inflammation, tissue remodeling, oxidative stress, and hypertrophic signaling pathways (Prasad and Quyyumi, 2004; Akhtar et al., 2020; Valentini et al., 2025). Contemporary understanding recognizes that the RAS extends beyond a simple binary opposition between the classical ACE/Ang II/AT1 receptor axis and the protective ACE2/Ang-(1–7)/Mas receptor pathway. The RAS is now conceptualized as a dynamic and highly integrated molecular network involving multiple bioactive peptides generated through different enzymatic processing pathways (see Figure 1), receptor subtypes (e.g. Mas and AT_2_R), and tissue-specific signaling mechanisms that collectively regulate inflammation, oxidative stress, fibrosis, vascular tone, and cellular remodeling (Bader et al., 2024; Jayawardena et al., 2026). In addition to canonical peptide generation through ACE and ACE2, other enzymes such as neprilysin and prolyl endopeptidases contribute to the formation of downstream angiotensin fragments with distinct biological effects. Furthermore, receptor signaling within the RAS exhibits considerable complexity, including ligand-specific signaling bias, receptor crosstalk, intracellular RAS components, and local tissue-restricted regulatory systems that function largely independently of the circulating endocrine RAS (Bader et al., 2024; Jayawardena et al., 2026). This expanded conceptual framework is particularly relevant in pulmonary disease, where localized dysregulation of tissue RAS signaling contributes to chronic inflammation, epithelial injury, fibroblast activation, and aberrant repair processes (Gan et al., 2023), thereby strengthening the rationale for therapeutic modulation of specific protective pathways such as Ang-(1–7).

Within this wider framework, one of the most extensively studied counter-regulatory interactions within the RAS is the balance between two major functional axes: the classical Angiotensin Converting Enzyme 1 (ACE1)/Ang II/AT1 receptor pathway, which mediates pro-inflammatory, profibrotic, and vasoconstrictive effects, and the opposing protective Angiotensin Converting Enzyme 2 (ACE2)/Ang-(1–7)/Mas receptor axis, which mediates anti-inflammatory, anti-fibrotic, and vasodilatory properties (Benter et al., 1993; 1995b; Akhtar et al., 2012; Akhtar et al., 2020; Santos et al., 2018). The seminal study by Santos et al. (2003) established the Mas receptor as the G protein-coupled receptor for Ang-(1–7), providing the molecular basis for the protective ACE2/Ang-(1–7)/Mas axis (Santos et al., 2003). Similarly, ACE2 was independently identified by Tipnis et al. (2000) and Donoghue et al. (2000) as an ACE homolog with distinct carboxypeptidase activity, establishing a critical alternative enzymatic pathway within the RAS (Donoghue et al., 2000; Tipnis et al., 2000). With regards to Ang-(1-7), it is primarily generated through ACE2-mediated degradation of Ang II or indirectly via sequential cleavage of Ang I, producing Ang-(1-9) by removing an amino acid from the C-terminus of Ang I and then yielding Ang-(1-7) via the enzymatic activity of neprilysin, thus providing a critical counter-regulatory mechanism within the RAS (Schwacke et al., 2013). Indeed, our group was the first to report that Ang-(1-7) behaves in a way opposite to that of Ang II and that IV administration of Ang-(1-7) produces blood pressure lowering effects by activating its own receptor (Benter et al., 1993). We also showed that chronic infusion of Ang-(1-7) using osmotic mini-pumps produces anti-hypertensive effects (Benter et al., 1995b; Benter et al., 1995a). Our studies in rabbit vascular smooth muscle cells showed that norepinephrine, through activation of adrenergic receptors, and Ang-(1-7), through activation of distinct type of angiotensin receptors, stimulate Ca (++)/calmodulin-dependent protein kinase II, which in turn, via mitogen-activated protein kinase activation, enhances cytosolic phospholipase A2 activity and release of arachidonic acid for prostaglandin synthesis (Muthalif et al., 1996; Muthalif et al., 1998). Antioxidant and anti-inflammatory effects of Ang-(1-7) were subsequently also shown by our group (Benter et al., 2008; Al-Maghrebi et al., 2009; Dhaunsi et al., 2010; El-Hashim et al., 2012; El-Hashim et al., 2019). Chronic treatment with Ang-(1–7) prevented activation of Tlr2, Ikbkb and Irak1, reduced NF-κB activity and downregulated the expression of C3, Il-6, Il-1β, Nalp12 and Casp1 in rat models of hypertension and/or diabetes (Al-Maghrebi et al., 2009). We later showed that chronic (4 weeks) IV delivery Ang-(1-7) or AVE 0991, a non-peptide analog of Ang-(1-7), using osmotic mini-pumps can prevent vascular, renal and cardiac dysfunction in a model of endothelial dysfunction and in models of hypertension and/or diabetes (Benter et al., 2006; 2007). Our studies also demonstrated that endogenous Ang-(1-7) inhibits diabetes-induced cardiac/renal NOX activity and end-organ damage by showing that chronic treatment with DX600, a selective ACE2 inhibitor, exacerbated diabetes-induced increase in cardiac and renal NOX activity (Yousif et al., 2012).

The evolving conceptualization of RAS, reflected by a better understanding of the mechanistic insight into ligand-specific enzymatic cascades and receptor-mediated signaling dynamics, positions it as a promising target for therapeutic intervention across various diseases, especially those within the respiratory domain like asthma and PF (Gan et al., 2023; Akhtar et al., 2020). Notably, the epidermal growth factor receptor (EGFR), a key transducer of cellular growth and inflammatory signals, can be aberrantly activated via angiotensin II-mediated transactivation, highlighting an important crosstalk between the renin–angiotensin system (RAS) and EGFR signaling (Akhtar et al., 2012; Shraim et al., 2021; Tawengi et al., 2024). This intersection not only amplifies pro-inflammatory and fibrotic responses but also reinforces the potential role of RAS dysregulation in the progression of pulmonary diseases, including asthma and PF (El-Hashim et al., 2012; Akhtar et al., 2020).

In pulmonary physiology, RAS serves as a pivotal determinant as its dysregulation is closely linked to asthma pathogenesis. RAS regulation has also been implicated in fibrotic lung diseases such as PF, where overactivation of the ACE/Ang II/AT1R axis contributes to epithelial apoptosis, fibroblast proliferation, as well as excessive extracellular matrix deposition (Meng et al., 2014). Chronic RAAS activation in COPD, particularly under hypoxic conditions, enhances Ang II/AT1R signaling, driving inflammation, oxidative stress, and airway remodeling. These effects are worsened by ACE2 downregulation in smokers, which depletes Ang-(1-7) and shifts the balance toward unchecked injury (Vasileiadis et al., 2018; Su et al., 2024). Similarly, in COVID-19, ACE2 internalization after viral entry causes Ang II accumulation and Ang-(1-7) loss, triggering vascular inflammation and fibrosis (Malhotra et al., 2020; Akhtar et al., 2020). The shared RAS dysregulation in COPD and COVID-19 underscores the therapeutic relevance of restoring the Ang-(1-7)/Mas axis, supported by early plasma therapy trials (Kuriakose et al., 2021; Onal et al., 2022). Taken together, the involvement of RAS dysregulation in COPD and COVID-19, both of which exhibit Ang-(1-7) depletion and benefit from its restoration, further strengthens the rationale for targeting the Ang-(1-7)/Mas axis in asthma and PF, where similar inflammatory and fibrotic mechanisms prevail.

This review examines the role and therapeutic potential of Ang-(1-7) in treating asthma and PF. In addition, and unlike previous reviews, we provide a comprehensive evaluation of its human safety profile based on published clinical trial data, focusing on its tolerability, reported side effects, dose dependency, and various routes of administration, while also highlighting advances in nanotechnology-based formulations that can enhance its stability, bioavailability, and targeted delivery for potential clinical use in lung diseases.

## 2 Role of Ang-(1–7) in lung diseases

### 2.1 Role of Ang-(1-7) in asthma

The therapeutic potential of Ang-(1-7) in asthma has been extensively examined in preclinical settings, encompassing both *in vivo* asthma models (e.g., ovalbumin-sensitized mice) and *in vitro* asthma-related systems (e.g., BEAS-2B bronchial epithelial cells and airway smooth muscle cells). Across these asthma models, Ang-(1-7) demonstrated the capacity to modulate key asthma pathophysiological processes, including airway inflammation, structural remodeling, airway hyperresponsiveness (AHR), and impaired lung function (see Table 1 for a summary of animal studies in asthma).

**TABLE 1:** Preclinical studies of angiotensin-(1-7) in experimental asthma and allergic airway inflammation.

#	Study	Animal model	Sample size	Intervention (dose, route)	Timing	Key findings
1	El-Hashim et al. (2012)	Male BALB/c mice; OVA-induced asthma (single sensitization + alum; intranasal challenge ×4 days)	DRC 6–16/group; A779 study 11–18/group; histology 6/group	Ang-(1-7) 0.03–0.3 mg/kg IP, 30 min before and 1 h after each challenge ×4 days	Preventative/concurrent	Dose-dependent reduction in BALF total cells, eosinophils, lymphocytes and neutrophils (macrophages unaffected); reduced peribronchial/perivascular inflammation, fibrosis and goblet-cell hyperplasia; lowered lung p-ERK1/2 and p-IκB-α; inhibited PHA-induced HPBMC proliferation *in vitro*. All effects A779-reversible (Mas-dependent); first report of an anti-asthmatic action of Ang-(1-7). Eosinophils ∼3→0.7 ×10^5^/mL; HPBMC proliferation ∼33% lower
2	Rodrigues-Machado et al. (2013)	Male BALB/c mice; chronic OVA asthma (4 sensitizations; nebulized challenge 3×/wk, days 21–51)	CTRL 17, OVA 21, treated 21; subgroups 4–7	AVE 0991* 1 mg/kg/day SC, days 21–54	Preventative (concurrent)	Reduced serum IgE and BAL/lung IL-5 (to control) and raised BAL IL-10 (A779-blocked); reduced airway, vascular and epithelial smooth-muscle thickness; restored bronchial contractile response; prevented right-ventricular hypertrophy; lowered lung Ang II. Validates pharmacological targeting of the Mas axis. IgE 154→51 A.U.; BAL IL-10–60→90 pg/mL
3	Magalhães et al. (2015)	Male BALB/c mice; chronic OVA asthma (4 sensitizations; nebulization 3×/wk, days 21–46)	CTRL/OVA/treated 25 each; subgroups 4–7	Ang-(1-7) 1 μg/h SC osmotic minipump ×28 days (days 21–49)	Preventative (concurrent)	Reduced BALF total cells and serum IgE; lowered lung IL-4, IL-5, GM-CSF, CCL2 and CCL5; normalized airway, parenchymal and perivascular inflammation; reduced collagen deposition and collagen I/III mRNA, epithelial thickening, AHR and vascular remodeling; prevented right-ventricular hypertrophy; lowered lung p-ERK1/2 and p-JNK. Mas receptor identified in bronchial epithelium and smooth muscle. BALF cells 38→18 ×10^5^/mL; IgE 12.5→7.0 ng/mL
4	Magalhães et al. (2018)	Male BALB/c mice; OVA-induced asthma (2 sensitizations + alum; 8 intranasal challenges)	CTRL/OVA 15 each; oral-treated 15; intranasal 10; assays 5–6	Single Ang-(1-7)/HPβCD, oral 60 μg/kg or intranasal 30 μg/kg, 24 h post-final challenge	Therapeutic (peak eosinophilic)	Reduced BAL eosinophils and lung EPO (mononuclear cells unchanged); increased eosinophil apoptosis (caspase-3) and macrophage efferocytosis; suppressed eosinophil NF-κB and lung p-IκB-α, p-ERK1/2 and GATA3; reduced ECM and collagen I/III mRNA. First demonstration of the Mas receptor on murine and human eosinophils; intranasal route achieved comparable resolution at half the oral dose. Eosinophils 10→3 ×10^5^/BAL; efferocytosis 3.8→15.0%
5	El-Hashim et al. (2019)	Male BALB/c mice; OVA-induced asthma (single sensitization + alum; intranasal challenge ×4 days)	BALF/histology 9–14; AHR 12–19; IF 4–8	Ang-(1-7) 0.3 mg/kg IP, 1 h before and 1 h after each challenge ×4 days	Preventative/concurrent	Reduced BALF total cells, lymphocytes, neutrophils and eosinophils (macrophages unaffected); attenuated peribronchial/perivascular inflammation, fibrosis and goblet-cell hyper/metaplasia; reduced AHR and *ex vivo* granulocyte chemotaxis; lowered lung p-Src, p-EGFR and p-ERK1/2 (all A779-reversible). Mechanism: blockade of Src-mediated EGFR transactivation. p-EGFR ∼48% lower; p-ERK1/2–78% lower
6	Magalhães et al. (2020)	Male BALB/c mice; chronic OVA asthma (4 sensitizations + 12 nebulized challenges over 4 weeks)	CTRL 15, OVA 15, treated 10; assays 4–6	Inhaled Ang-(1-7)/HPβCD 4.5 µg/dose twice daily ×14 days (days 35–48)	Therapeutic (established)	Reduced plasma IgE, BAL total cells, eosinophils and mononuclear cells; lowered lung IL-5 and CCL11; attenuated peribronchial infiltrate; reversed remodeling with reduced ECM and collagen deposition and partial epithelial thinning; reduced MMP-9 expression and activity and MMP-12. ECM 18→11%; collagen 19→10%
7	Magalhães et al. (2021)	BALB/c mice; OVA/LPS mixed eosinophilic and neutrophilic asthma	Assays 5–6; CTRL 11, OVA + LPS 13, treated 13	Oral Ang-(1-7)/HPβCD 60 μg/kg, two doses (1 h and 7 h post-LPS)	Therapeutic (peak inflammation)	Restored exploratory and locomotor activity; normalized respiratory rate, tidal volume and minute ventilation; reduced BALF total cells, neutrophils and eosinophils (mononuclear cells unchanged) and lung EPO and MPO; reduced mucus and ECM deposition and lung p-ERK1/2. Rearings restored from ∼10 toward control (∼26); locomotion restored
8	Xu et al. (2023)	Male C57BL/6 mice; OVA + chronic intermittent hypoxia (OSA comorbidity model)	6/group	Ang-(1-7) 150 μg/kg/week IP, days 14–42 (concurrent with CIH)	Therapeutic (concurrent)	Reduced lung injury and fibrosis; lowered BALF IL-6, IL-1β, IL-17A and TNF-α; reduced EMT markers (α-SMA, collagen I/IV, vimentin, Snail) and restored E-cadherin. Mechanism: lower HIF-1α reduces THBS1–BECN1 interaction, suppressing autophagy and EMT. α-SMA and collagen IV reduced; E-cadherin increased
9	Zhou et al. (2023)	Male WT and ATG5^−/−^ C57BL/6J mice; OVA-induced asthma	5/group	Intranasal Ang-(1-7) 30 μg/kg/day ×10 days from day 8	Preventative	Reduced airway resistance; lowered BALF IL-4, IL-5 and IL-33; reduced inflammatory infiltration, α-SMA and TGF-β1; reduced LC3B and Beclin-1. Effects mechanistically linked to inhibition of ATG5-mediated autophagy and phenocopied in ATG5^−/−^ mice. IL-4 ∼40→22 pg/mL; infiltration ∼16→8%
10	Magalhães et al. (2024)	Male and female BALB/c mice; OVA eosinophilic asthma (secondary OVA/LPS rechallenge)	6–8/group	Single oral Ang-(1-7)/HPβCD 60 μg/kg, 24 h post-final challenge	Therapeutic (post-peak)	A single dose accelerated resolution (lower BAL total cells and eosinophils by 48 h; lower lung IL-4/IL-13 by 72 h) and reprogrammed the lung toward a pro-resolving phenotype by 120 h (higher IL-10, CD4^+^Foxp3^+^ Tregs and resolution-phase macrophages with enhanced efferocytosis); pretreatment protected against secondary OVA and heterologous LPS rechallenge (reduced eosinophils, ECM and FeNO). Durable pro-resolving phenotype for ≥5 days
11	Gonçalves et al. (2025)	Male BALB/c mice; OVA-induced allergic asthma	7–12/group	Ang-(1-7)/HPβCD oral 60 μg/kg/day or intranasal 30 μg/kg/day, concurrent with 8-day challenge	Therapeutic	Reduced lung inflammation and ameliorated anxiety- and depression-like behaviours (elevated plus maze, open field, tail suspension); intranasal route lowered prefrontal-cortex TNF-α and IL-6; both routes raised IL-10, supporting lung-brain axis regulation. Lung inflammation 43→∼30%; immobility 65→24 s (intranasal)

Studies are ordered chronologically. *AVE, 0991 is a synthetic non-peptide Mas receptor agonist used as an Ang-(1-7) mimetic. Arrows (→) denote change from model/vehicle toward the treated value. All abbreviations are defined in Supplementary Table S1.

One of the central features of asthma is chronic airway inflammation, typically driven by Th2 and Th17 immune responses, with infiltration of eosinophils, neutrophils, and lymphocytes (Banno et al., 2020). The Th2 axis, characterized by cytokines such as IL-4, IL-5, and IL-13, promotes IgE production, eosinophilic recruitment, and mucus hypersecretion, whereas the Th17 subset, through release of IL-17A, IL-17F, and IL-22, contributes to neutrophilic inflammation, airway remodeling, and steroid resistance (Banno et al., 2020; Zhou et al., 2023). Elevated IL-17 levels correlate with asthma severity and corticosteroid non-responsiveness in both clinical and experimental models. In this inflammatory context, Ang-(1–7), the heptapeptide generated predominantly through ACE2-mediated degradation of Ang II, exerts broad anti-inflammatory effects via binding to the Mas receptor, a G-protein–coupled receptor expressed in pulmonary tissues (Benter et al., 1993) (Figure 1). Activation of this receptor triggers intracellular pathways such as the PI3K/AKT/eNOS axis, promoting nitric oxide (NO) production, smooth muscle relaxation, and improved airway tone. In OVA-induced murine asthma, Ang-(1–7) administration significantly attenuated bronchoalveolar lavage fluid (BALF) inflammatory cell counts, particularly eosinophils and neutrophils (El-Hashim et al., 2019; Magalhães et al., 2021), while other studies demonstrated decreased levels of IL-4, IL-5, coupled with increases in the anti-inflammatory IL-10 cytokine (Rodrigues-Machado et al., 2013). These reductions indicate that Ang-(1–7) interferes with upstream Th2 polarization and cytokine transcription, possibly through inhibition of GATA3, ERK1/2, and IκB-α signaling (Magalhães et al., 2018). Moreover, Ang-(1–7) downregulates NF-κB activation and inflammasome activity (El-Hashim et al., 2012; Sun et al., 2017), suppresses Th2 cytokines via inhibition of the MAPK/ERK axis (El-Hashim et al., 2012; El-Hashim et al., 2019), and activates phosphatases such as DUSP1 and MKP-1 (Gava et al., 2009; McCollum et al., 2012). These actions limit transcription of pro-inflammatory genes and impair priming of the NLRP3 inflammasome, reducing secretion of IL-1β. By upregulating phosphatases, Ang-(1–7) also deactivates ERK and p38 MAPK pathways, preventing sustained pro-inflammatory signaling and promoting resolution of airway inflammation. Collectively, these findings indicate that Ang-(1–7) exerts broad anti-inflammatory effects through modulation of multiple signaling pathways involved in allergic airway inflammation.

Genetic deficiency of the Mas receptor aggravates allergic pulmonary inflammation, increasing eosinophilic infiltration, Th2 cytokine production, ERK1/2 activation, extracellular matrix deposition, and airway remodeling, thereby providing direct evidence for the endogenous protective role of the Ang-(1–7)/Mas axis in asthma (Magalhães et al., 2016). Consistent with these findings, pharmacological inhibition of Mas receptor signaling produces a similar loss of protection. Blockade of the receptor abolishes the anti-inflammatory and anti-remodeling benefits of aerobic exercise in experimental asthma, including reductions in IgE levels, neutrophilic infiltration, and structural airway changes (Gregório et al., 2021). Together, these genetic and pharmacological studies confirm that intact Mas receptor signaling is required for the protective effects of the Ang-(1–7) pathway in the asthmatic lung. Importantly, the actions of Ang-(1–7) appear to extend beyond classical anti-inflammatory suppression toward active promotion of inflammation resolution. In addition to reducing pro-inflammatory cytokine signaling, Ang-(1–7) has been shown to promote eosinophil apoptosis, enhances alveolar macrophage efferocytosis, and supports resolution-phase immune reprogramming, emphasizing its role as a pro-resolving immunomodulatory peptide (Magalhães et al., 2018). To further support the pro-resolving role of Ang-(1–7), another study demonstrated that inhaled Ang-(1–7)/HPβCD not only reduced eosinophilic inflammation but also reversed established airway remodeling, decreasing collagen deposition and MMP-9/MMP-12 expression in a chronic asthma model (Magalhães et al., 2020). More recently, oral or intranasal administration of an Ang-(1–7) cyclodextrin formulation attenuated pulmonary inflammation and reduced associated anxiety- and depressive-like behaviors in a mouse model of allergic pulmonary inflammation (Gonçalves et al., 2025). Together, this positions Ang-(1–7) as a counter-regulatory mediator capable of restoring immune balance predominantly in Th2-driven asthma, with emerging but less direct evidence for modulation of Th17-associated pathways (Finkelman et al., 2010; El-Hashim et al., 2012).

A particularly impactful mechanism of Ang-(1-7) involves the inhibition of EGFR signaling, which is implicated in both airway inflammation and remodeling (Liu Y et al., 2023; El-Hashim et al., 2019). Ang II transactivates EGFR via Src-dependent phosphorylation, initiating ERK1/2 and p38 MAPK cascades. However, Ang-(1-7) blocks this process through Mas receptor-dependent inhibition of Src kinase activity, thereby preventing EGFR autophosphorylation at critical tyrosine residues (e.g. Y992, Y1068, Y1086, Y1148) (Akhtar et al., 2012; Akhtar et al., 2015). This was clearly demonstrated in allergic murine models, whereby Mas receptor activation significantly suppressed EGFR and ERK1/2 phosphorylation, along with this effect being reversed by the Mas receptor antagonist A779 (El-Hashim et al., 2019). Due to the suppression of EGFR and ERK1/2 phosphorylation, Ang-(1-7) attenuates mucus hypersecretion, epithelial thickening, and fibroblast activation, which are key remodeling features that drive airflow obstruction and steroid resistance in chronic asthma. Moreover, these upstream effects translate into downstream functional benefits, whereby Ang-(1-7) robustly attenuated allergen-induced AHR, showing anti-AHR efficacy comparable to corticosteroids but without systemic side effects, reinforcing its potential value in steroid-resistant asthma (El-Hashim et al., 2019). Along with this, the heptapeptide promoted eosinophil apoptosis, enhanced alveolar macrophage efferocytosis, and suppressed key regulators of allergic inflammation such as GATA3 and IκBα (Magalhães et al., 2018). Collectively, these multifaceted actions support the role of Ang-(1-7) as a promising disease-modifying agent in asthma, capable of targeting both inflammation and remodeling pathways.

Building upon the anti-remodeling effects, in OVA-induced murine asthma models, Ang-(1-7) significantly attenuated peribronchial and perivascular fibrosis, suppressed subepithelial collagen deposition, and prevented thickening of the extracellular matrix (El-Hashim et al., 2019; Xu et al., 2023; Magalhães et al., 2024). These are hallmarks of irreversible airway remodeling and progressive airflow limitation, as evidenced by histological analysis that revealed reductions in goblet cell hyperplasia, which limits mucus overproduction, and smooth muscle hypertrophy, decreasing the potential for bronchoconstriction, thus directly preserving airway function (Banno et al., 2020; Gan et al., 2023). These structural benefits offered by Ang-(1-7) are underscored by the suppression of pro-fibrotic signaling pathways, particularly the TGF-β/SMAD2/3 axis, with the downregulation of TGF-β1 expression and SMAD2/3 phosphorylation (Gan et al., 2023). Therefore, as a consequence of this suppression, there is a marked reduction in fibroblast activation, myofibroblast differentiation, and collagen synthesis, as well as disruption of the transcriptional machinery that drives ECM expansion. Furthermore, Ang-(1-7) inhibits NOX4-derived oxidative stress, a critical upstream mediator of fibrogenesis that promotes reactive oxygen species (ROS) generation and redox-sensitive gene expression involved in remodeling (Meng et al., 2015; Sheng et al., 2024). As a result of this inhibition, Ang-(1-7) disrupts the oxidative activation of the TGF-β1–Smad2/3 fibrogenic pathway, thereby preventing fibroblast-to-myofibroblast transition and epithelial–mesenchymal signaling that underlie airway thickening and remodeling in chronic asthma.

In addition to this, Ang-(1-7) suppresses ECM-degrading enzymes MMP-9 and MMP-12, which are typically upregulated in chronic asthma and contribute to severe tissue remodeling (Magalhães et al., 2020). Through this suppression, Ang-(1-7) mitigates excessive proteolytic degradation of collagen and elastin, protects against emphysematous changes, and thus preserves alveolar structure, supporting its role as a disease-modifying agent in advanced asthma. More mechanistic insights were demonstrated in IL-13–stimulated BEAS-2B (a bronchial epithelial cell line) and Human Bronchial Smooth Muscle Cells (HBSMC), where Ang-(1-7) significantly reduced ATG5 protein expression in a dose-dependent manner, indicating its role in modulating autophagic activity (Xu et al., 2023). Along with this, there was a marked reduction in IL-25 and IL-13 secretion from BEAS-2B cells, as well as decreased TGF-β1 and α-SMA protein levels in HBSMC, whereby these effects were closely mimicked by ATG5 siRNA, indicating that Ang-(1–7)’s anti-inflammatory and anti-fibrotic actions are at least partially mediated by downregulation of ATG5. On the other hand, overexpression of ATG5 through cDNA transfection reversed these effects, restoring high levels of IL-25, IL-13, TGF-β1, and α-SMA, even in the presence of Ang-(1–7), which confirms ATG5’s role as a critical upstream regulator. These findings confirm that Ang-(1-7) attenuates IL-13–induced epithelial cytokine release and smooth muscle fibrotic transformation by targeting the ATG5-dependent autophagy axis, a pathway that contributes to airway remodeling and chronic disease progression in asthma. Beyond local airway effects, emerging evidence suggests that Ang-(1–7) may indirectly modulate AT1 receptor-associated signaling in a context-dependent manner, potentially shifting downstream responses away from classical pro-fibrotic Gq/PLC signaling toward more cytoprotective pathways involving ERK, AKT, and Src kinases (Kim et al., 2012; Balakumar and Jagadeesh, 2014; Delaitre et al., 2021; Singh and Karnik, 2024). However, direct classification of Ang-(1–7) as a canonical biased AT1 receptor agonist remains less firmly established than for Ang II or synthetic ligands, and these actions of Ang-(1-7) warrant further study.

Functionally, Angiotensin-(1-7) demonstrated significant restorative effects on lung function in asthmatic models, shown by marked increases in tidal volume and minute ventilation (Magalhães et al., 2021), alongside reductions in airway resistance, lung elastance, and enhanced dynamic compliance (El-Hashim et al., 2019). These improvements point to enhanced airflow and parenchymal function, suggesting an anti-bronchoconstrictor/bronchodilatory role and indirect benefits via tissue remodeling suppression. Additionally, Ang-(1-7) inhibited the phosphorylation of pro-inflammatory and pro-fibrotic mediators such as ERK1/2, NF-κB, Src kinase, and EGFR, which are central to asthma pathogenesis and progression (El-Hashim et al., 2019). The large reduction in ERK1/2 phosphorylation of greater than 50% reflects potent molecular inhibition that was reversed by the Mas receptor antagonist, A779, confirming the Mas receptor-dependent specificity of this effect (El-Hashim et al., 2019). In brief, the suppression of this signaling disrupts downstream cytokine production, matrix deposition, and smooth muscle proliferation, offering mechanistic support for the use of Ang-(1-7) as a therapeutic for asthma.

Lastly, a dose-dependent pattern has been observed in many studies when assessing the therapeutic efficacy of Ang-(1-7) in preclinical asthma models. Higher doses (1 μM) were associated with larger reductions in airway inflammation and fibrosis, as opposed to lower doses (0.1 μM) that produced milder but still significant effects on pro-inflammatory cytokine suppression and airway remodeling (Zhou et al., 2023). Similarly, this trend was reflected at a molecular level, whereby dose-dependent inhibition of ERK1/2 and NF-κB activation, two key signaling pathways in asthma pathogenesis, was observed (El-Hashim et al., 2019). Another study that used escalating doses of Ang-(1-7) (0.03, 0.1, and 0.3 mg/kg, intraperitoneally) led to a significant reduction in BALF total cell counts, eosinophils, lymphocytes, and neutrophils in OVA-induced asthmatic mice (El-Hashim et al., 2012). Collectively these studies demonstrate the critical role of dosing in determining the extent of anti-inflammatory, anti-fibrotic, and anti-remodeling effects. Optimizing the dose is essential to balance efficacy and safety, highlighting the need for future clinical dose-ranging studies that aim to establish optimal therapeutic regimens for Ang-(1-7) in asthma (see also Section 3).

### 2.2 Role of Ang-(1–7) in pulmonary fibrosis

PF represents a complex interplay between chronic inflammation, profibrotic signaling, oxidative injury, and aberrant tissue remodeling (Khalfaoui and Pabelick, 2023; Nell et al., 2026). While it is classically associated with interstitial lung diseases such as IPF, fibrotic remodeling is also a prominent feature of severe steroid-resistant asthma, manifesting as airway wall thickening, collagen deposition, and fixed airflow obstruction. In both contexts, fibrosis contributes substantially to disease severity, therapeutic refractoriness, and recurrent, potentially life-threatening exacerbations (Khalfaoui and Pabelick, 2023). Given the overlapping cellular mechanisms particularly those involving fibroblast activation, epithelial–mesenchymal transition (EMT), and oxidative stress, the anti-fibrotic potential of Ang-(1–7) has gained growing attention across both PF and asthma. A series of preclinical studies in rat and murine models have consistently demonstrated its broad-spectrum anti-fibrotic actions, providing strong translational evidence of pharmacological and therapeutic relevance (see Table 2).

**TABLE 2:** Preclinical studies of angiotensin-(1-7) in experimental pulmonary fibrosis and related fibrotic lung injury.

#	Study	Animal model	Sample size	Intervention (dose, route)	Timing	Key findings
1	Shenoy et al. (2010)	Male Sprague-Dawley rats; single IT BLM (2.5 mg/kg) → PF (parallel MCT-PH study)	4–6/group (PF arm)	Lentiviral Ang-(1-7) or ACE2 (3 × 10^8^ TU), intratracheal, 2 weeks before BLM.	Preventative	Lentiviral Ang-(1-7) (and ACE2) overexpression reduced Ashcroft score and lung hydroxyproline and prevented associated pulmonary hypertension and right-ventricular hypertrophy; downregulated TGF-β mRNA and AT1R protein while restoring depleted ACE. In the companion MCT study A779 abolished protection (Mas-dependent); systemic blood pressure unchanged. Ashcroft ∼5→3.5; RVSP ∼38→22 mmHg
2	Chen et al. (2013)	Male C57BL/6 mice; single IT LPS (5 mg/kg) → acute lung injury and early PF (days 3 and 7)	5/group per timepoint	Ang-(1-7) 100 ng/kg/min SC from 1 h pre-LPS ×3 or 7 days; comparators losartan, A779	Preventative	Reduced lung-injury score, pulmonary edema, Ashcroft score, hydroxyproline and collagen I/III; lowered TGF-β and p-Smad2/3. Efficacy comparable to losartan; A779 alone worsened fibrosis (endogenous Mas tone protective). Inflammation peaked at day 3 while fibrosis progressed to day 7. Hydroxyproline ∼25% lower
3	Meng et al. (2014)	Male Wistar rats; single IT BLM (5 mg/kg) → PF (28 days); HFL-1 and primary fibroblasts *in vitro*	6/group (×4–5 groups)	Ang-(1-7) or Ang II 600 μg/kg/day SC ×28 days from BLM; lentiACE2 2 weeks pre-BLM.	Preventative (concurrent)	Ang-(1-7)/ACE2 reduced Ashcroft score, Masson density and hydroxyproline and lowered CTGF, α-SMA and collagen I; rebalanced RAS (lower ACE/AT1R, higher ACE2/Mas) and inhibited MAPK (ERK/p38/JNK) and NF-κB signalling, while Ang II exacerbated fibrosis; *in vitro* Ang-(1-7) restored fibroblast apoptosis. Context-dependent dual role: Ang-(1-7) alone (no insult) was pro-fibrotic and pro-inflammatory, abolished by irbesartan. A779-reversible (Mas-dependent)
4	Meng et al. (2015)	Male Wistar rats; single IT BLM (5 mg/kg) → PF; primary lung fibroblasts *in vitro*	12/group (×4–5 groups)	Ang-(1-7) or Ang II 600 μg/kg/day SC ×28 days from BLM; lentiACE2 2 weeks pre-BLM.	Preventative (concurrent)	Ang-(1-7)/ACE2 reduced Ashcroft score, Masson density and hydroxyproline and lowered CTGF, α-SMA and collagen I; markedly rebalanced RAS toward the ACE2/Mas axis; selectively reduced NOX4 and H_2_O_2_ and switched off the RhoA/Rho-kinase pathway, while Ang II exacerbated every readout. Identified Rac1–NOX4 coupling and a context-dependent dual role; Ang-(1-7) effects A779-reversible. ACE2/ACE ratio ∼34-fold higher; Mas/AT1R ∼62-fold higher
5	Zambelli et al. (2015)	Male Sprague-Dawley rats; HCl aspiration + high-stretch ventilation (acute ARDS) and 2-week late ARDS.	Series-dependent (late ARDS: vehicle 7, treated 8)	IV Ang-(1-7) (early 6.5 or 1440 μg/kg/day ×5 h; rescue 100 μg/kg/h ×3 h from 2 h post-injury) or SC 300 μg/kg/day ×2 weeks (late)	Both (early concurrent, rescue, late)	Improved PaO_2_/FiO_2_ at both early doses and on delayed rescue; reduced peripheral and alveolar leukocytes without changing BAL cytokines or permeability; late SC infusion reduced acid-injured-lung hydroxyproline at 2 weeks. The cleanest in-table illustration of a post-injury rescue window. Rescue PaO_2_/FiO_2_ ∼365→445; late hydroxyproline ∼42% lower
6	Sun et al. (2017)	Male Wistar rats; single IT BLM (5 mg/kg) → PF; primary lung fibroblasts *in vitro*	12/group (×6 groups)	Ang-(1-7) or Ang II 600 μg/kg/day SC ×28 days from BLM; lentiACE2 2 weeks pre-BLM.	Preventative (concurrent)	Ang-(1-7)/ACE2 reduced Ashcroft score, hydroxyproline and septal thickening; lowered lung miR-21, p-ERK, nuclear NF-κB, NLRP3 and collagen I while raising Spry1, whereas Ang II exacerbated fibrosis. *In vitro*, anti-miR-21 reproduced all protective effects, identifying miR-21 as the obligate mediator and placing ACE2/Ang-(1-7) upstream of miR-21 transcription. Ashcroft ∼4.5→2.8
7	Pan et al. (2018)	Male Wistar rats; passive cigarette smoke ×8 weeks → smoking-related PF; primary fibroblasts + CSE *in vitro*	12/group (×3 groups)	Ang-(1-7) ∼600 μg/kg/day SC infusion throughout the 8-week exposure	Therapeutic (concurrent)	Reduced Ashcroft score, collagen deposition, hydroxyproline and α-collagen I; lowered NOX4 and lung H_2_O_2_; restored autophagic flux (reduced p62/LC3-II accumulation, increased autolysosomes) by rescuing cathepsin B/D function. Mechanism: NOX4/ROS-driven block of autophagic degradation drives collagen synthesis; all *in vitro* effects A779-reversible. Hydroxyproline ∼11→6 μg/mL; Ashcroft ∼4.0→2.2
8	Cao et al. (2019)	Male Sprague-Dawley rats; LPS three-hit regimen → early PF following ARDS.	6/group (×6 groups)	Ang-(1-7) 600 μg/kg/day IP ×20 days from day 3; comparators AVE0991, A779	Therapeutic (early, post-injury)	Reduced lung-injury and Ashcroft scores; lowered plasma/BALF TGF-β and BALF Ang II while raising plasma Ang-(1-7); inhibited EMT (higher E-cadherin, lower vimentin); rebalanced membrane RAS (lower AT1R, higher Mas) and reduced p-Src. Effects reproduced by AVE0991 and reversed by A779 (Mas-dependent). Plasma TGF-β ∼5,700→2,300 pg/mL
9	Gao et al. (2019)	Male Wistar rats; IT or inhaled silica → silicotic PF; Ang II-stimulated fibroblasts; human silicosis cohort	6/group; human n = 60	SC Ang-(1-7) 25 μg/kg/day ×12 weeks from wk 12 (plus Ac-SDKP arms)	Therapeutic (concurrent)	Reduced silicotic nodule number and area and lung collagen I and α-SMA; raised meprin-α (the Ac-SDKP synthetase). Established a cooperative ACE2/Ang-(1-7)/Mas-Ac-SDKP loop confirmed pharmacologically (ACE2 activator raised, inhibitor lowered meprin-α). In patients, serum ACE correlated inversely and ACE2/Ang-(1-7) positively with FEV_1_. Effects A779-reversible. Lung collagen I markedly reduced
10	Rago et al. (2019)	Male C57BL/6J mice; single intranasal BLM (3 mg/kg) → PF.	5–7/group per arm	Oral Ang-(1-7)/HPβCD 60 μg/kg twice daily; preventive (1 h pre-BLM) and therapeutic (day 3, 7 or 14 start)	Both (preventative and three therapeutic windows)	Preventive treatment improved survival, reduced BAL cellularity and collagen deposition and improved lung function, with increased early neutrophil apoptosis (pro-resolution); a day-3 start gave partial benefit; day-7 and day-14 starts gave none. Defines a narrow therapeutic window in which efficacy is lost once fibrosis is established. Preventive survival ∼65→90%; fibrosis score ∼6→3.5
11	Zhang et al. (2019)	Male Wistar rats; inhaled silica ×24 weeks → silicotic PF; MRC-5 fibroblasts; human pneumoconiosis specimens	10/group (×6 groups)	SC Ang-(1-7) 576 μg/kg/day ×8 weeks from wk 16 (plus Ac-SDKP/captopril arms)	Therapeutic (established; ongoing exposure)	Reduced silicotic nodule number and area and lung collagen I and α-SMA and improved breathing pattern; suppressed Hedgehog signalling (lower SHH, SMO, GLI1, GLI2; higher PTC, GLI3) in lung and Ang II-stimulated fibroblasts. *In vitro*, GLI1 (not SMO) inhibition blocked fibroblast transition, indicating non-canonical, SMO-independent GLI1 activation. The authors note this Hedgehog finding is silica/coal specific. Nodule area ∼17→11%
12	Sheng et al. (2024)	Male C57BL/6 mice; single IT BLM (5 mg/kg) → PF; TGF-β1-stimulated fibroblasts	Small per-figure cohorts	Ang-(1-7) 600 μg/kg/day IP ×21 days from BLM; comparators AVE0991, A779	Preventative (concurrent)	Attenuated weight loss and improved survival; reduced Szapiel/Ashcroft scores, hydroxyproline and α-SMA/collagen I/III; lowered TGF-β1, TNF-α and IL-6 and raised IL-10; reduced ROS/MDA and raised antioxidant enzymes. Mechanism: restored Nox4-Nrf2 redox homeostasis (lower Nox4/Keap1, higher nuclear Nrf2, HO-1, NQO1). Effects reproduced by AVE0991 and reversed by A779. Serum IL-10–185→310 pg/mL

Studies are ordered chronologically. Arrows (→) denote change from model/vehicle toward the treated value. Lentiviral ACE2 arms are included where they were used as the Ang-(1-7)-generating intervention. All abbreviations are defined in Supplementary Table S1.

One important proposed antifibrotic mechanism underlying Ang-(1–7)’s activity involves suppression of NADPH oxidase 4 (NOX4)-derived ROS, which are key drivers of fibroblast activation, migration, and extracellular matrix (ECM) production. The heptapeptide reduces NOX4 expression and ROS accumulation, thereby inhibiting the RhoA/ROCK pathway, a signaling axis essential for myofibroblast contractility and cytoskeletal remodeling (Meng et al., 2015; Sheng et al., 2024). Functionally, this translates into decreased collagen deposition and preservation of epithelial structures in silica- and cigarette smoke–induced lung injury models. Ang-(1–7) further restores redox balance by modulating the NOX4–Nrf2 axis, alongside effects on autophagy-related signaling. Notably, the effects of Ang-(1–7) on autophagy appear to be context-dependent. Zhou et al. demonstrated that Ang-(1–7) attenuates airway remodeling in chronic intermittent hypoxia (CIH)-aggravated asthma by suppressing excessive autophagic activity through the HIF-1α/THBS1/BECN1 axis, as evidenced by normalization of LC3B-II and p62 levels and reduced autophagosome/autolysosome formation (Zhou et al., 2023). In contrast, Pan et al. reported that cigarette smoke-induced pulmonary fibrosis was characterized by impaired autophagic flux, reflected by the accumulation of both LC3B-II and p62, and showed that Ang-(1–7) restored functional autophagy by reducing these markers (Pan et al., 2018). Collectively, these findings suggest that Ang-(1–7) may modulate autophagy in a context-dependent manner, contributing to its protective effects across chronic lung diseases. Since defective autophagy and persistent oxidative imbalance drive progressive fibrosis, these actions are particularly relevant to PF. The upregulation of antioxidant defenses, including superoxide dismutase (SOD), and the preservation of mitochondrial integrity further disrupt ROS-driven positive feedback loops (Sheng et al., 2024). Collectively, these effects highlight Ang-(1–7)’s therapeutic potential in conditions where oxidative stress and impaired autophagy underlie fibrotic progression.

Beyond redox control, Ang-(1–7) exerts potent anti-inflammatory and pro-resolving effects through inhibition of MAPK and NF-κB signaling. In bleomycin-induced models, treatment reduced collagen synthesis, suppressed fibroblast proliferation, and enhanced myofibroblast apoptosis (Meng et al., 2014). Promoting fibroblast susceptibility to apoptosis is especially critical for halting progressive fibrosis and limiting irreversible remodeling. A key contributor to fibrotic remodeling is EMT. Ang-(1–7) suppresses EMT through multiple mechanisms: inhibition of NOX-derived H_2_O_2_ accumulation, blockade of NLRP3 inflammasome activation and IL-1β signaling, as well as attenuation of TGF-β-induced SMAD2/3 phosphorylation (Meng et al., 2014; Sun et al., 2017; Sheng et al., 2024). By dampening oxidative stress and inflammasome activity, Ang-(1–7) prevents redox-sensitive amplification of TGF-β signaling and downregulates mesenchymal markers such as α-SMA and fibronectin, preserving epithelial integrity. In addition, Ang-(1–7) antagonizes EGFR transactivation, a major EMT driver via ERK1/2 and TGF-β crosstalk (Akhtar et al., 2012; El-Hashim et al., 2019). By interrupting this feed-forward loop, it blunts sustained ERK1/2 activation, thereby reducing fibroblast accumulation and ECM deposition. In silica-induced models, Ang-(1-7) also modulated the Hedgehog pathway, suppressing Sonic Hedgehog (SHH), smoothened (SMO), and GLI1/2, while upregulating PTC and GLI3, shifting signaling toward a repressive state that limits mesenchymal reprogramming (Zhang et al., 2019). Within this specific silica-induced fibrosis model, Hedgehog pathway modulation by Ang-(1–7) was associated with reduced profibrotic transcriptional activity and decreased myofibroblast persistence (Zhang et al., 2019), effects consistent with experimental Hedgehog inhibition. However, whether this mechanism is broadly applicable across other fibrosis models remains to be established.

The Mas receptor is a key mediator of the antifibrotic actions of Ang-(1–7). Upon binding to Mas, Ang-(1–7) suppresses several profibrotic signaling cascades, including ERK1/2 and p38 MAPK, through activation of phosphatases such as SHP-1 (Gava et al., 2009; El-Hashim et al., 2012; Meng et al., 2014). These effects limit fibroblast proliferation, myofibroblast differentiation, and airway smooth muscle remodeling, thereby reducing the cellular processes that drive progressive fibrosis. Further support for the importance of Mas signaling comes from studies using the non-peptide Mas agonist AVE0991, which similarly reduced α-SMA expression and extracellular matrix deposition (Sheng et al., 2024). In addition to regulating MAPK signaling, Ang-(1–7) attenuates Src-dependent EGFR transactivation, a pathway implicated in abnormal vascular and airway remodeling, further reinforcing its antifibrotic and tissue-protective effects (Akhtar et al., 2012; El-Hashim et al., 2019). Ang-(1–7) also stimulates eNOS, enhancing NO production and promoting vasodilation (Kuriakose et al., 2021), mechanisms particularly relevant in advanced PF complicated by vascular remodeling and pulmonary hypertension (Shenoy et al., 2010).

Upon Mas receptor binding, Ang-(1–7) inhibits Hedgehog signaling (via GLI3 repressor dominance), restores autophagy and mitophagy, reduces oxidative and inflammatory stress, and promotes epithelial barrier integrity and vascular function (Zhang et al., 2019). It also shifts macrophage polarization toward an anti-inflammatory M2 phenotype, collectively fostering lung repair and protection. A complementary mechanism supported by preclinical evidence is that Ang-(1–7) may indirectly modulate AT1 receptor-associated signaling in a context-dependent manner, potentially shifting downstream responses away from classical pro-fibrotic signaling toward more cytoprotective pathways. However, direct classification of Ang-(1–7) as a canonical β-arrestin-biased AT1 receptor agonist requires further validation (Kim et al., 2012; Singh and Karnik, 2024). Nonetheless, this possible dual receptor interaction, combining Mas receptor and biased AT1R signaling actions, implies Ang-(1–7) has the capacity to act as a multifunctional modulator capable of simultaneously suppressing profibrotic signaling and enhancing reparative responses. Preclinical studies further support these phenotypic effects: in bleomycin-induced fibrosis, early oral administration of Ang-(1–7) significantly reduced lung inflammation, collagen accumulation, and mortality while improving pulmonary compliance (Rago et al., 2019). However, delayed intervention (≥7 days post-injury) failed to reverse established fibrosis, suggesting that timing of administration is critical to fully harness its therapeutic benefits. Moreover, Ang-(1–7) decreased hydroxyproline content, right ventricular hypertrophy, and pulmonary arterial pressure, reinforcing its systemic cardiopulmonary benefits (Shenoy et al., 2010).

**FIGURE 2:**
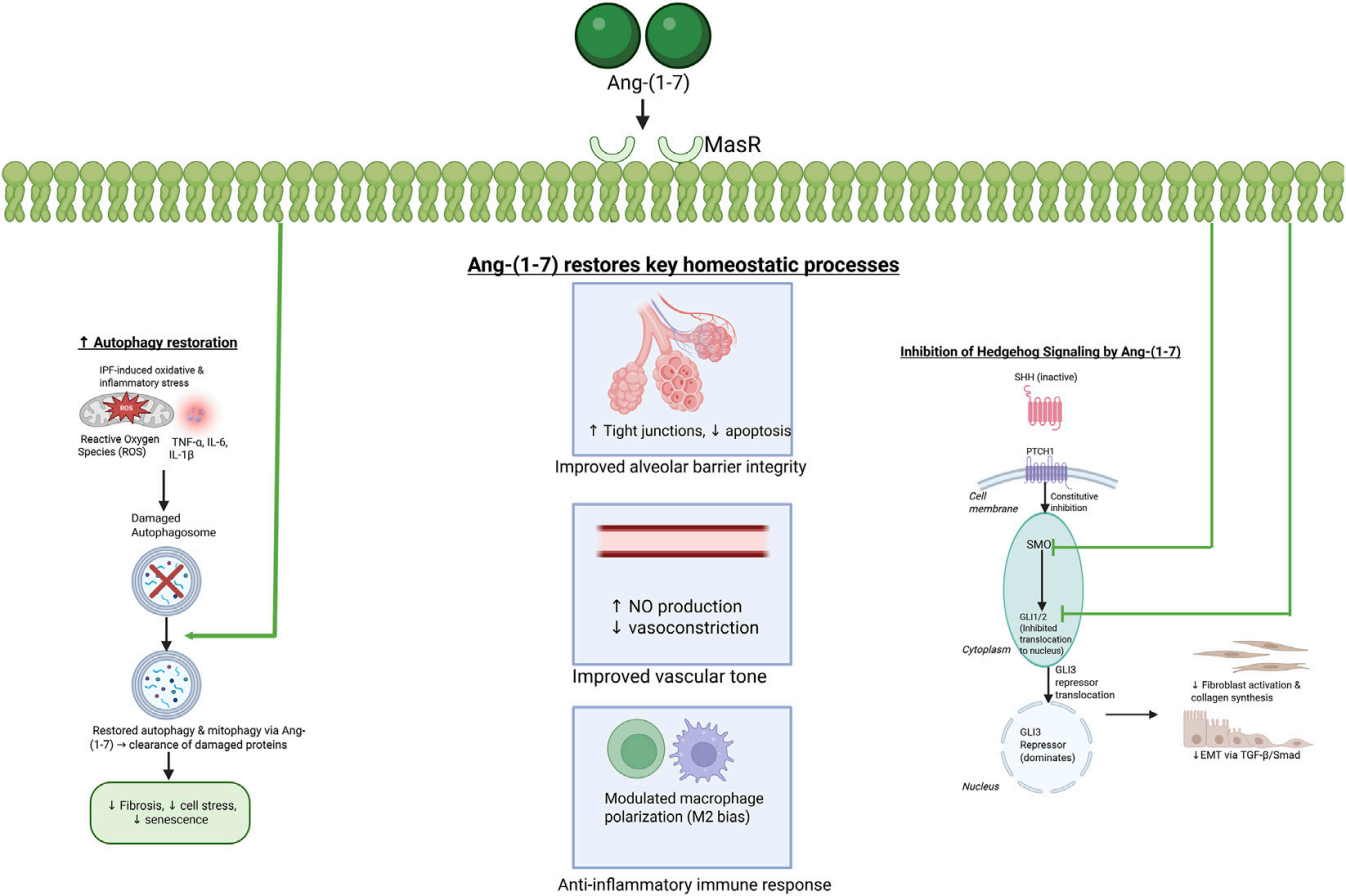
Mechanisms of Antifibrotic Action of Angiotensin-(1–7) in PF. Ang-(1–7) exerts antifibrotic effects in PF through multiple mechanisms: inhibition of Hedgehog signaling (via GLI3 repressor dominance), restoration of autophagy and mitophagy to reduce oxidative stress and clear damaged proteins, and promotion of key homeostatic processes such as improved alveolar barrier integrity, enhanced vascular tone, and M2 macrophage polarization. Together, these pathways reduce fibroblast activation, collagen synthesis, inflammation, and fibrosis while preserving lung tissue integrity. Well-established mechanisms, including restoration of alveolar barrier integrity, vascular homeostasis, and immune modulation, are emphasized centrally in large text, whereas autophagy restoration and Hedgehog pathway modulation are emerging and/or model-specific mechanisms for which current evidence base remains comparatively limited are depicted as smaller sized text.

Ang-(1–7) mitigates PF through a coordinated network of mechanisms involving suppression of oxidative stress, inflammation, EMT, fibroblast persistence, model-specific profibrotic signaling pathways (including Hedgehog signaling), and autophagy disruption. By restoring epithelial integrity, vascular tone, and immune homeostasis (see also Figure 2), Ang-(1–7) emerges as a promising, multi-targeted therapeutic candidate for fibrotic lung conditions such as PF and severe asthma. However, the translational weight of these preclinical findings is bounded by a heavy reliance on bleomycin-induced model of PF, which fundamentally reflects an acute, inflammatory-driven, and often self-resolving lung injury rather than the chronic, progressive, and irreversible pathophysiology of human PF (Kolb et al., 2020). Thus, further research in clinically relevant models is warranted as well as studies designed to optimize delivery, timing, and dosing in clinical settings to fully realize Ang-(1-7)’s translational potential.

## 3 Chemical modifications and nanotechnology-based formulations of Ang-(1–7) for potentially improving drug delivery in the clinic

The clinical translation of Ang-(1–7), a bioactive heptapeptide of the protective renin–angiotensin system axis, has been limited by rapid proteolytic degradation, a short circulating half-life, poor oral bioavailability, and restricted penetration across physiological barriers (Aghazadeh-Habashi and Khajehpour, 2021). Native Ang-(1–7) exhibits a plasma half-life of approximately 20–30 min and oral bioavailability below 2% (Mordwinkin et al., 2012), necessitating frequent or continuous administration to maintain therapeutic exposure. These pharmacokinetic constraints have driven the development of chemical modifications and nanotechnology-based delivery platforms aimed at enhancing peptide stability, prolonging systemic exposure, and enabling targeted tissue delivery. A dedicated overview of various delivery strategies in respiratory disease contexts is provided in Table 3.

**TABLE 3:** Summary of key studies utilizing drug-delivery systems and formulation strategies for angiotensin-(1-7) in respiratory disease.

#	Study	Delivery system	Sample size	Formulation	Dose, route and frequency	Model and study type	Pharmacokinetics/stability	Efficacy and safety
1	Supé et al. (2016)	Normal saline (IV)	98 male Sprague-Dawley rats, split controls, injury-only, and 4 treatment time-window groups (n = 8 or more/group)	Ang-(1-7) in 0.9% NaCl for IV infusion	50 pmol/kg/min continuous IV across four time windows (pretreatment, continuous post-injury, short-term, late)	Oleic-acid-induced acute lung injury in male Sprague-Dawley rats; multi-arm time-window study	Short plasma half-life (20–30 min) requires continuous infusion; saline stability not characterized	Continuous post-injury infusion (30–240 min) gave optimal protection: reversed lung wet/dry ratio, BALF protein, MPO, TNF-α and pulmonary vascular resistance and raised the lung ACE2/ACE activity ratio. Late treatment did not protect barrier function but still reduced MPO. No adverse effects in uninjured controls; small non-significant fall in arterial pressure
2	Magalhães et al. (2018)	Cyclodextrin (HPβCD)	55 male BALB/c mice, split into 4 groups (control, asthma-only, and two Ang-(1–7) treatment groups (n = 10 or more/group)	Ang-(1-7)/HPβCD inclusion complex; dual oral (water) and intranasal (saline) formulations; foundational HPβCD platform for this group	Single dose at peak inflammation (24 h post-final challenge); oral 60 μg/kg or intranasal 30 μg/kg	Male BALB/c mice; OVA-induced asthma. Therapeutic (established)	HPβCD enables oral and intranasal delivery; PK not measured	Both routes reduced BAL eosinophils and lung EPO (mononuclear cells unchanged); increased eosinophil apoptosis and efferocytosis (15.0% vs. 3.8%); suppressed eosinophil NF-κB and lung p-IκB-α, p-ERK1/2 and GATA3; reduced ECM and collagen I/III mRNA. First demonstration of the Mas receptor on murine and human eosinophils; intranasal achieved comparable resolution at half the oral dose. Safety NR.
3	Zhang et al. (2018)	Mini-osmotic pump (SC)	40 C57BL/6J mice (20 male, 20 female), split into 4 groups: air control, cigarette smoke only, and smoke with Ang-(1–7) treatment (n = 5 or more/group)	Ang-(1-7) in saline delivered via SC osmotic pump	0.3 mg/kg SC continuous infusion ×2 weeks, after 8-week smoke exposure	Male and female C57BL/6J mice; cigarette-smoke COPD. Therapeutic (established)	PK not detailed; sustained delivery assumed	Reduced BAL total cells, neutrophils and lymphocytes (macrophages unaffected); lowered lung/BALF KC and TNF-α (IL-6 unchanged); reduced lung fibrosis (Masson, BALF TGF-β1) and bronchial inflammation; activated bronchial Mas1 and lowered p-IκBα and nuclear NF-κB-p65. No effect on impaired lung function or emphysema. Safety NR.
4	Magalhães et al. (2020)	Cyclodextrin (HPβCD), inhaled	40 male Balb/c mice, split into 3 groups: control, asthma-only, and asthma + inhaled Ang-(1–7) treatment (n = 10 or more/group)	Ang-(1-7)/HPβCD in saline by ultrasonic nebulization; free peptide was ineffective by inhalation, so encapsulation was required	4.5µg + 6.9 µg HPβCD per dose, inhaled twice daily ×14 days	Male BALB/c mice; chronic OVA asthma. Therapeutic (established)	HPβCD enabled stable inhaled delivery; PK not measured (prior oral Cmax ∼6 h)	Reduced plasma IgE, BAL total cells, eosinophils and mononuclear cells and lung IL-5/CCL11; reversed remodeling (ECM 18→11%, collagen 19→10%, partial epithelial thinning); reduced MMP-9 expression and activity and MMP-12. Safety NR.
5	Alabsi et al. (2021)	Dry powder inhaler	Cell-line Study	Co-spray-dried Ang-(1-7) or glycopeptide PNA5 with trehalose (25:75); amorphous glassy micro/nanoparticles	*In vitro* aerosol testing at 60 L/min (Neohaler); cell viability 0.1–1000 μg/mL; no *in vivo* dose	Physicochemical characterization plus 2D/3D human airway cell models. *In vitro*	High glass-transition temperature (∼143 °C–155 °C) and low residual water confirm amorphous stability; PK not assessed	Respirable aerosol suitable for deep-lung deposition (Ang-(1-7):trehalose FPF ∼50%, MMAD 1.70 µm; PNA5:trehalose FPF ∼32%, MMAD 2.09 µm). No loss of viability across nasal, brain and lung cell models at all concentrations; chronic *in vivo* safety NR.
6	Magalhães et al. (2021)	Cyclodextrin (HPβCD)	37 male BALB/c mice, split into 3 groups: control, OVA + LPS challenge, and OVA + LPS + oral Ang-(1–7) treatment (n = 11 or more/group)	Ang-(1-7)/HPβCD inclusion complex in water; oral gavage	60 μg/kg, two oral doses 6 h apart (1 h and 7 h post-LPS)	BALB/c mice; mixed eosinophilic/neutrophilic OVA/LPS asthma. Therapeutic (established)	HPβCD enables oral delivery with gastric protection; PK not measured	Restored exploratory and locomotor activity; normalized respiratory rate, tidal volume and minute ventilation; reduced BAL total cells, eosinophils and neutrophils (mononuclear cells unchanged) and lung EPO/MPO; reduced mucus and ECM and lowered lung p-ERK1/2 (*in vivo* MAPK evidence). Safety NR.
7	Magalhães et al. (2024)	Cyclodextrin (HPβCD)	Male and female BALB/c mice, split into control, OVA challenge, and OVA + oral Ang-(1–7) groups (n = 5 or more/group)	Ang-(1-7)/HPβCD inclusion complex in water; oral gavage	60 μg/kg single oral dose, 24 h post-final challenge	Male and female BALB/c mice; OVA asthma with secondary rechallenge. Therapeutic (established)	HPβCD enables oral bioavailability; PK not quantified. Durable single-dose effect for ≥5 days	A single dose accelerated resolution (lower BAL cells/eosinophils by 48 h; lower lung IL-4/IL-13 by 72 h) and reprogrammed the lung toward a pro-resolving phenotype by 120 h (higher IL-10, CD4^+^Foxp3^+^ Tregs and macrophage efferocytosis); protected against secondary OVA and heterologous LPS rechallenge. Safety NR.
8	Mendes et al. (2024)	Liposomes (intranasal)	K18-hACE2 transgenic mice (mixed sex), n = 7–8/group, split into 5 groups: liposomal Ang-(1–7), free Ang-(1–7), saline control, empty liposomes, and remdesivir (n = 7 or more/group)	Ang-(1-7) in soybean-phosphatidylcholine liposomes (∼100 nm, PDI 0.018, EE 13.4%); 15 µL/nostril	0.73 mg/kg intranasal every 12 h × 5 days, from 8 h post-infection	K18-hACE2 mice infected with SARS-CoV-2 gamma variant. Therapeutic (early post-infection)	Sustained *in vitro* release, half-time 9.3 h (vs. free peptide released by 3 h)	Prolonged median survival (6 vs. 5 days); 3–4-log reduction in lung viral load; reduced lung IL-6 (∼65%) and TNF (∼70%); reduced myeloid and inflammatory monocyte infiltration; no effect on brain/kidney viral load. Empty-liposome control showed no vehicle toxicity
9	Gonçalves et al. (2025)	Cyclodextrin (HPβCD)	Male Balb/c mice (8–10 weeks old), split into groups: control, asthma-only, asthma + oral Ang-(1–7), and asthma + intranasal Ang-(1–7); with additional intracerebroventricular infusion subgroup (n = 7 or more/group)	Ang-(1-7)/HPβCD inclusion complex (oral: water; intranasal: saline)	Oral 60 μg/kg or intranasal 30 μg/kg daily ×8 days during challenge	Male BALB/c mice; OVA-induced allergic asthma. Preventative (concurrent)	HPβCD enhanced stability and slow release; PK not quantified	Both routes reduced lung inflammatory infiltrate (oral 31%, intranasal 29% vs. 43%) and improved anxiety- and depression-like behaviours; intranasal route lowered prefrontal-cortex TNF-α and IL-6; both raised IL-10. Safety NR.
10	Magalhães et al. (2025)	Cyclodextrin (HPβCD)	Male C57BL/6 mice (8 weeks old), split into groups: control, elastase-induced emphysema (EIPE, and EIPE + oral Ang (1–7) treatment (n = 5 or more/group)	Ang-(1-7)/HPβCD inclusion complex; oral gavage	60 μg/kg + 92 μg/kg HPβCD daily ×4 weeks	Male C57BL/6 mice; elastase-induced emphysema. Therapeutic (established)	HPβCD protected the peptide and enabled sustained release; PK not detailed	Reduced lung collagen deposition and restored alveolar architecture; downregulated collagen I/III, MMP9 and VEGF; suppressed inflammation (NF-κB1, NLRP3, IL-1β; p-NF-κB) and senescence (p53, p16, p21); reactivated Wnt/β-catenin repair signalling. Safety NR.

Studies are ordered chronologically. “Both” under study type indicates the same study tested preventative and therapeutic dosing. NR, not reported. All abbreviations are defined in Supplementary Table S1.

### 3.1 Chemical modification and sustained Mas receptor activation

Chemical modification strategies to enhance the biological stability of Ang-(1–7) while preserving its biological activity include substitution with non-natural amino acids (e.g., cis-3-(aminomethyl) cyclobutane carboxylic acid) (Wester et al., 2017), cyclization via a thioether bridge (Kluskens et al., 2009), C-terminal amidation, and glycosylation (Hay et al., 2019; Hoyer-Kimura et al., 2021). Among these approaches, C-terminally glycosylated Ang-(1–7), termed PNA5 (Ang-1-6-O-Ser-Glc-NH_2_), has emerged as particularly promising from a therapeutic standpoint in preclinical models of CNS disease (Hay et al., 2019; Hoyer-Kimura et al., 2021). Glycosylation significantly enhances biological stability, extending peptide half-life by up to four-fold through steric hindrance of proteolytic degradation (Hay et al., 2019; Hoyer-Kimura et al., 2021). Although most investigations of PNA5 have focused on neuroprotection and vascular cognitive impairment, the mechanistic importance of sustained Mas receptor signaling is highly relevant to asthma and PF, where chronic inflammation, oxidative stress, and extracellular matrix remodeling are central drivers of disease progression.

Encapsulation of PNA5 within poly (lactic-co-glycolic acid) (PLGA) nanoparticles further optimizes its pharmacokinetic profile, enabling prolonged Mas receptor activation and sustained downstream signaling (Encinas-Basurto et al., 2022). These findings underscore the importance of maintaining continuous Ang-(1–7) exposure to achieve durable disease modification rather than transient symptomatic effects. Moreover, PNA5 formulated with trehalose into nanoparticle-based dry powder inhalers for targeted respiratory delivery has also been developed (Encinas-Basurto et al., 2022), representing a particularly attractive strategy for asthma and PF therapy.

### 3.2 Nanoparticle platforms: general principles and lung targeting

Beyond chemical modification, nanotechnology-based drug delivery platforms offer versatile solutions to overcome the intrinsic limitations of peptide therapeutics (Kheraldine et al., 2021). Nanoparticles (NPs) can traverse epithelial and endothelial barriers through transcytosis and paracellular transport, enabling delivery to otherwise inaccessible tissues (Sheth et al., 2021; Yuan et al., 2024). Nanoencapsulation also protects Ang-(1–7) from enzymatic degradation and delays renal clearance, thereby prolonging systemic exposure (Encinas-Basurto et al., 2022).

Nanoparticles exhibit intrinsic passive targeting properties, accumulating preferentially in reticuloendothelial system (RES)-rich organs, including the lungs, liver, and spleen, through opsonization-mediated phagocytic uptake (Sousa De Almeida et al., 2021; Lee et al., 2024). When excessive RES sequestration limits bioavailability, surface modification strategies such as PEGylation or ligand conjugation (e.g., antibodies or peptides) can be employed to extend circulation time and enhance active targeting (Sheth et al., 2021). Importantly, pathological lung conditions such as asthma and PF are associated with increased vascular permeability, which may further promote nanoparticle accumulation through enhanced permeability and retention (EPR)-like effects (Fang et al., 2004; Qiao et al., 2021).

### 3.3 Pulmonary delivery: inhalable nano-formulations

For asthma and PF, localized pulmonary delivery represents a particularly attractive therapeutic strategy, allowing direct targeting of the diseased organ while reducing systemic exposure and required dose (He X et al., 2025). Pulmonary administration exploits the lung’s large surface area and dense vascular network that avoids hepatic first-pass metabolism, facilitating efficient drug absorption and rapid onset of action (He X et al., 2025; Li et al., 2022).

Although Ang-(1–7) nano-delivery has not yet been evaluated directly in asthma or PF models, compelling preclinical evidence demonstrates effective lung targeting using inhalable nanotechnology-based formulations (He X et al., 2025; Li et al., 2022). Liposomal Ang-(1–7) systems (Mendes et al., 2024) and spray-dried inhalable particles (Alabsi et al., 2021) exemplify this approach, combining nanoscale encapsulation with optimized aerodynamic properties to enhance peptide stability, prolong mucosal residence, and enable sustained local release.

An intranasal liposomal Ang-(1–7) formulation composed of soybean phosphatidylcholine produced flexible, near-neutral vesicles (∼100 nm; PDI < 0.1) with an encapsulation efficiency of 13.4% (Mendes et al., 2024). This system exhibited sustained *in vitro* release (half-life ∼9.3 h) and, in SARS-CoV-2–infected K18-hACE2 mice, significantly improved survival, reduced pulmonary viral load by 3–4 log units, and attenuated IL-6 and TNF-α production as well as leukocyte infiltration, outperforming free Ang-(1–7) and empty liposomal controls (Mendes et al., 2024). Complementarily, amorphous trehalose-based nanoparticles achieved deep lung deposition (fine particle fraction 32%–50%) and controlled dissolution, enhancing local pharmacodynamic effects while limiting systemic exposure (Alabsi et al., 2021). These studies collectively demonstrate that nanoparticle engineering directly modulates the pulmonary pharmacokinetics and pharmacodynamics of Ang-(1–7), with liposomal carriers extending peptide half-life and trehalose matrices optimizing deposition and dissolution kinetics.

However, long-term pulmonary safety remains an important consideration for chronic inhalation therapy, as repeated nanoparticle exposure may influence pulmonary homeostasis. With respect to immunogenicity, Ang-(1–7) is an endogenous seven-amino-acid peptide and has not been associated with localized allergic inflammation in preclinical studies, suggesting a low likelihood of anti-drug antibody formation (Mordwinkin et al., 2012; Gallagher et al., 2014). However, whether this favorable immunogenicity profile extends to other synthetic Mas receptor peptide-based agonists remains uncertain and warrants further investigation. Other pulmonary safety considerations also remain relevant, including macrophage uptake of inhaled particles, potential interference with mucociliary clearance, and the consequences of prolonged particle retention within the respiratory tract. In this context, although the minimal pulmonary concentration required for effective Mas receptor activation in asthma or PF models remains undefined, current nanoformulations are specifically engineered to prolong peptide stability, enhance lung deposition, and sustain local release. Platforms including PNA5-loaded PLGA nanoparticles, liposomal carriers, and spray-dried trehalose matrices therefore aim to maintain continuous local Mas receptor activation while minimizing systemic exposure. Ultimately, while repeated and sustained pulmonary delivery may be necessary to achieve therapeutic efficacy, it could also increase the risk of receptor desensitization and, in the case of synthetic Mas receptor agonists, potential immunogenicity. Additionally, the impact of the pulmonary delivery systems on long term safety in terms of immuno-, bio- and geno-compatibility (see also discussion in Section 3.7) remain to be fully ascertained and requires further evaluation in long-term safety studies within a disease-specific setting.

### 3.4 Translational precedent from inhaled peptide therapeutics

Translational insight can be drawn from FDA-approved inhaled therapies that have successfully overcome the inherent instability of peptide and protein drugs. Pulmonary delivery bypasses first-pass metabolism, enables ultra-rapid alveolar absorption, and allows tunable duration of action, albeit requiring careful dose optimization (Heinemann et al., 2017; Hirsch et al., 2024). Afrezza® (inhaled insulin) demonstrates that short-lived peptides can be reformulated into dry-powder aerosols for efficient alveolar deposition and rapid systemic uptake (Rendell, M., 2014; Fleming et al., 2015). Similarly, tobramycin inhalation powder (TOBI®) (Bonsignore, 1998; Rosalia et al., 2022) and treprostinil dry-powder inhaler (Tyvaso® DPI) (Spikes et al., 2022) illustrate how localized pulmonary delivery achieves high lung concentrations while minimizing systemic spillover. Together, these precedents establish a clear translational pathway for inhaled Ang-(1–7) formulations in asthma and PF.

### 3.5 Oral nano-formulations and cyclodextrin inclusion complexes

Despite the advantages of pulmonary delivery, oral administration remains the most desirable route for chronic therapy due to its convenience, non-invasiveness, and high patient compliance. However, oral delivery of Ang-(1–7) has historically been hindered by gastrointestinal enzymatic degradation and poor bioavailability. Recent advances in cyclodextrin inclusion complexes and complementary nanotechnological strategies have substantially advanced this field. Cyclodextrins (CDs) are cyclic oligosaccharides featuring a hydrophobic cavity that facilitates host–guest complexation, improving solubility, stability, and bioavailability of labile drugs. Their clinical safety is well established, with FDA-approved derivatives such as hydroxypropyl-β-cyclodextrin (HPβCD) and sulfobutylether-β-cyclodextrin (SBEβCD) used in antifungal agents (itraconazole) and neuromuscular blockade reversal (Sugammadex) (Jambhekar and Breen, 2016; Mirza et al., 2020; Cuoco et al., 2023).

HPβCD-based Ang-(1–7) formulations significantly enhance oral bioavailability by protecting the peptide from gastrointestinal degradation, resulting in a 12-fold increase in plasma levels within 6 h of administration (Lula et al., 2007; Marques et al., 2011). These formulations exert robust cardioprotective, anti-inflammatory, antifibrotic, and metabolic effects via Mas receptor activation, improving cardiac function, reducing infarct size, attenuating pulmonary remodeling in asthma, and restoring insulin signaling in metabolic syndrome (Santos et al., 2003; Figueiredo et al., 2019; Magalhães et al., 2020). It is noteworthy that HPβCD/Ang-(1–7) cyclodextrin formulations have already demonstrated functional, anti-inflammatory, and airway remodeling benefits in experimental models of allergic asthma, supporting their translational potential for chronic pulmonary diseases (Magalhães et al., 2018; 2020; 2021; 2024). In parallel, their sustained release properties and improved systemic exposure may enable once-daily dosing, further supporting the feasibility of these formulations as oral peptide therapeutics (Fraga-Silva et al., 2011). Also, HPβCD-mediated stabilization of Ang-(1–7) supports prolonged therapeutic activity in both peripheral and central inflammatory models (Gonçalves et al., 2025). Building on this, oral administration of an Ang-(1–7)/HPβCD formulation (60 μg/kg/day Ang-(1–7); 92 μg/kg/day HPβCD) promoted structural lung repair and reduced inflammatory and senescence markers in an elastase-induced emphysema model, although lung bioavailability was not directly quantified (Magalhães et al., 2025). Stable pharmacokinetics were maintained over 4 weeks despite the low daily dose, underscoring the formulation’s potency and suitability for chronic non-invasive therapy.

Additional refinements have included synergistic integration with lipid nanoparticles (LNPs), where HPβCD-Ang-(1–7) normalized the tumor microenvironment, reduced collagen I deposition, improved vascular perfusion, and enhanced nanoparticle penetration without detectable hepatic or renal toxicity (Da Silva et al., 2024). Prepared via freeze-drying at a 1:1 M ratio, this formulation preserved Ang-(1–7)’s antifibrotic and anti-angiogenic properties while extending oral bioavailability. Innovative oral strategies have also included plant-based bioencapsulation, wherein Ang-(1–7) was expressed in lettuce chloroplasts and delivered orally as lyophilized plant cells (Daniell et al., 2024). In this system, chloroplast-integrated DNA encoding CTB-ACE2 or CTB-Ang-(1–7) enabled peptide bioencapsulation, protecting against gastric degradation and facilitating intestinal release through bacterial enzymatic cleavage. A cholera toxin B (CTB) fusion tag enhanced absorption via GM1 receptor binding, resulting in a 56.5% increase in systemic exposure following repeated gavage, with no observed toxicity even at high doses (Daniell et al., 2024). This platform eliminated cold-chain requirements, enabled room-temperature stability, and supported scalable, patient-friendly oral delivery. Collectively, these oral nano-formulations significantly increase AUC, improve bioavailability, and reduce dosing frequency, which are key requirements for long-term management of chronic diseases such as PF and severe asthma.

### 3.6 Sustained-release systems and controlled delivery platforms

Across many preclinical studies, Ang-(1–7) has been administered in simple aqueous vehicles (e.g., saline) via subcutaneous (SC), intravenous (IV), intraperitoneal (IP), or intracerebral routes (Dhaunsi et al., 2010; Mordwinkin et al., 2012; Yousif et al., 2014; Supé et al., 2016; Mińczuk et al., 2025). While these approaches ensure immediate bioavailability, they expose the peptide to rapid proteolysis and require frequent or continuous dosing.

Sustained drug delivery approaches, such as implantable mini-osmotic pumps, represent a robust strategy to overcome Ang-(1–7)’s rapid clearance and poor oral bioavailability. Continuous zero-order infusion maintains steady-state plasma concentrations, avoiding peak-trough variability associated with bolus dosing. This strategy has demonstrated efficacy across multiple disease models, including unilateral ureteral obstruction–induced renal fibrosis (Zimmerman et al., 2015), diabetic cardiomyopathy (Mori et al., 2014), and oleic acid–induced acute lung injury (Supé et al., 2016). In PF and asthma models, continuous low-dose infusion reduced collagen deposition, myofibroblast differentiation, airway hyperresponsiveness, and inflammation through sustained Mas receptor activation (Rodrigues-Machado et al., 2013; Mori et al., 2014; Magalhães et al., 2015; Supé et al., 2016). However, sustained or high-concentration delivery of Ang-(1–7) may also induce adaptive signaling changes and Mas receptor desensitization through classical GPCR regulatory mechanisms. Prolonged receptor stimulation has been shown to trigger rapid clathrin-mediated Mas receptor internalization into early endosomal compartments, thereby attenuating downstream signaling responsiveness (Gironacci et al., 2011). Additional pharmacological studies further support the presence of atypical desensitization and adaptive signaling responses following continuous Mas receptor activation (Tirupula et al., 2014). Accordingly, sustained-release Ang-(1–7) strategies may require careful dose optimization or intermittent delivery approaches to balance prolonged therapeutic activity with preservation of receptor responsiveness. Although macroscopic, osmotic pumps embody controlled-release principles foundational to advanced nanotechnologies, such as PEGylated liposomes, polymeric nanoparticles, and peptide hydrogels, that may further refine lung targeting and pharmacodynamic precision.

Among biodegradable carriers, poly (lactic-co-glycolic acid) (PLGA) has emerged as a clinically validated platform, with FDA-approved formulations including leuprolide acetate, goserelin acetate, and risperidone (Okada, 2011; Qi et al., 2019; Li et al., 2025). PLGA nano- and microparticles encapsulating glycosylated Ang-(1–7) as PNA5 achieved sustained release over 14 days with encapsulation efficiencies exceeding 50% (Encinas-Basurto et al., 2022). Acid-terminated PLGA nanoparticles (∼350 nm) and microparticles (∼2.4 µm) demonstrated superior loading and minimal burst release, extending PNA5’s serum half-life beyond that of the free peptide and reducing dosing frequency from daily to biweekly. Dual-phase hydrogels (Zhang et al., 2024) and nano-in-micro PLGA systems (Subbiah et al., 2015) further highlight the importance of spatiotemporal release control and aerodynamic suitability for inhaled lung-targeted therapy. Given their aerodynamic suitability and enzymatic protection, these systems present a compelling rationale for development as inhalable dry powders for lung-targeted therapy in asthma and PF.

### 3.7 Dendrimers as bioactive delivery platforms

Polyamidoamine (PAMAM) dendrimers offer an additional class of nanocarriers with highly controlled architecture and tunable surface chemistries (Kheraldine et al., 2021; Alamos-Musre et al., 2025). G4-PAMAM dendrimers exhibit strong peptide binding and pH-dependent release, protecting Ang-(1–7) from ACE and DPP3 cleavage and extending predicted half-life by more than two-fold (Chi et al., 2022). Acidic microenvironments promote peptide release, enabling targeted unloading at inflamed or diseased sites. Synergistic integration of PAMAM dendrimers with continuous infusion systems further stabilizes Ang-(1–7), enhancing residence time and therapeutic efficacy in models of muscle atrophy and functional decline (Chi et al., 2022). Such properties are highly relevant to chronic lung diseases, where sustained and localized Ang-(1–7) activity may be required to suppress airway remodeling.

Notably, drug delivery systems are increasingly recognized not merely as passive carriers but as biologically active entities capable of modulating gene expression and cell-signaling pathways (Akhtar and Benter, 2007; Hollins et al., 2007; Kheraldine et al., 2021). Accordingly, optimizing PAMAM dendrimers for clinical application necessitates careful assessment of both genotoxicity and broader biological compatibility. Cationic PAMAM dendrimers have been shown to induce generation- and surface-dependent transcriptomic alterations, including NF-κB–mediated downregulation of cell-cycle–associated genes and modulation of EGFR signaling in multiple cells (Hollins et al., 2007; Kheraldine et al., 2021). While such bioactive effects underscore the need for rigorous safety screening, they may also confer therapeutic advantages. Indeed, our group has demonstrated that PAMAM dendrimers function as modulators of EGFR-dependent cell-signaling cascades both *in vitro* and *in vivo* (Akhtar et al., 2013; 2015; 2016b; 2016a; 2019). For example, chronic administration of cationic PAMAM dendrimers in a rat model of diabetes inhibited the EGFR–ERK1/2–ROCK signaling axis, resulting in attenuation of diabetes-induced vascular remodeling and endothelial dysfunction (Akhtar et al., 2019). Consistent with these findings, PAMAM dendrimers have exhibited synergistic anti-inflammatory, antimicrobial, and gene-modulatory properties, supporting their role as active co-therapeutic agents rather than inert delivery vehicles (Chauhan et al., 2009; Chis et al., 2020; Kheraldine et al., 2021).

The intrinsic ability of PAMAM dendrimers to suppress EGFR signaling (Akhtar et al., 2013; Akhtar et al., 2016a) is particularly relevant in asthma, where EGFR overactivation contributes to airway remodeling, mucus hypersecretion, and chronic inflammation, and where Ang-(1–7)–mediated EGFR inhibition has been shown to exert therapeutic benefit (El-Hashim et al, 2017; El-Hashim et al, 2019). This dual functionality provides a compelling rationale for the development of inhaled PAMAM–Ang-(1–7) formulations that integrate targeted peptide delivery with complementary gene-modulatory effects. Nonetheless, chronic exposure to PAMAM dendrimers has also been associated with adverse cardiovascular effects, including impaired cardiac function following ischemia–reperfusion injury (Babiker et al., 2020; Akhtar et al., 2025). Importantly, these deleterious effects were mitigated by co-administration of Ang-(1–7), which restored cardiac function and attenuated injury-associated perturbations (Akhtar et al., 2022).

**FIGURE 3:**
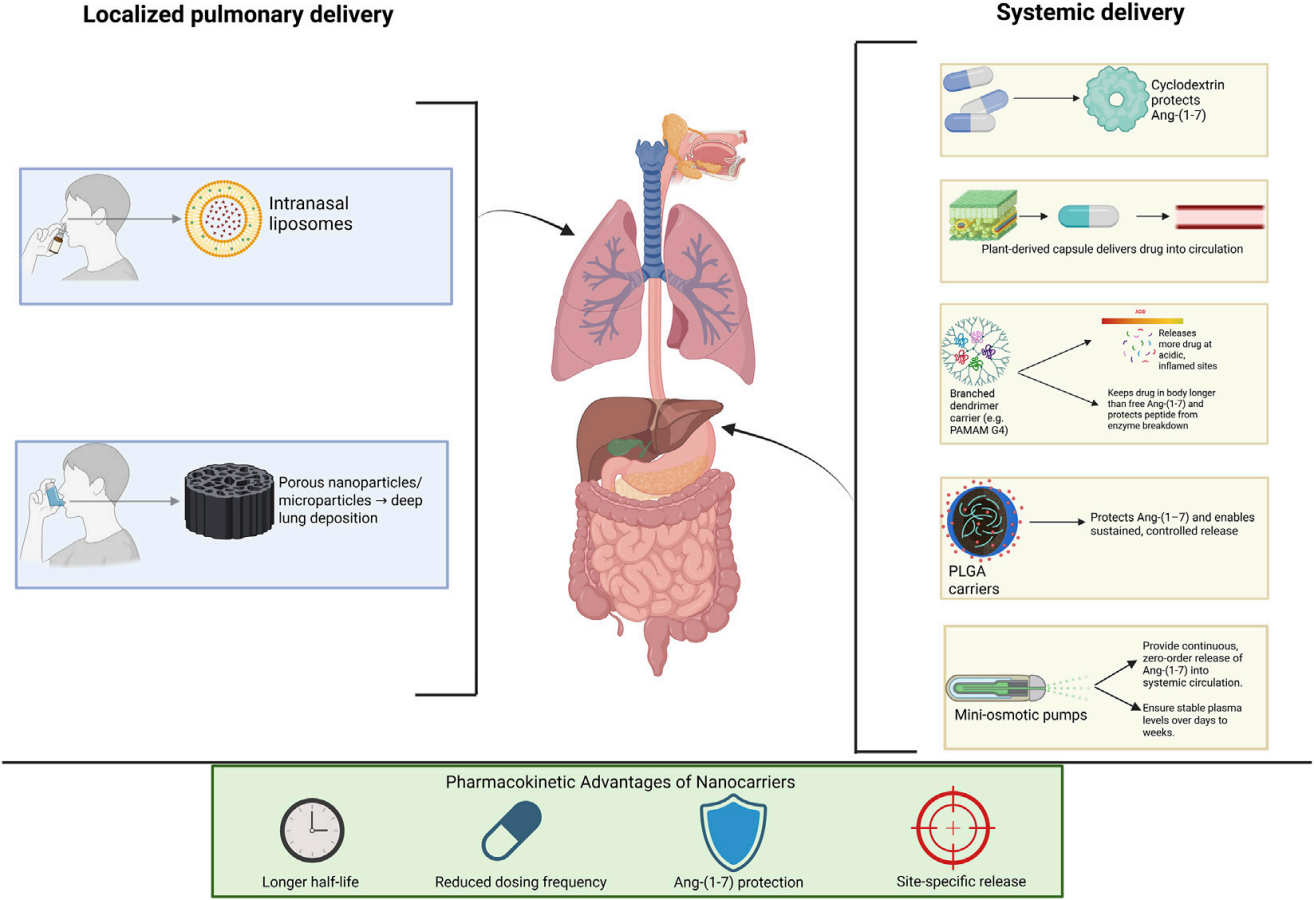
Nanotechnology-Based Formulations of Ang-(1–7) for Improved Drug Delivery. Nanocarrier strategies for Ang-(1-7) delivery include localized pulmonary approaches (intranasal liposomes, dry powder inhalers, PLGA nano/microparticles) and systemic methods (cyclodextrin inclusion complexes, plant bioencapsulation, dendrimers), whereby these formulations protect the peptide from enzymatic degradation, prolong circulation or lung residence, enable sustained and site-specific release, and reduce dosing frequency. To reflect the current state of evidence, cyclodextrin-based formulations represent the most extensively characterized delivery platforms, whereas pulmonary liposomes, dry powder inhalers, dendrimers, and plant-based bioencapsulation remain comparatively early-stage and are supported by more limited data. Collectively, they enhance Ang-(1-7) pharmacokinetics and therapeutic potential in asthma and PF.

Collectively, advances in chemical modification, nano-encapsulation, and sustained-release technologies have reshaped the pharmacological profile of Angiotensin-(1–7), extending its half-life from minutes to hours or days, enhancing tissue penetration, and enabling precise spatial and temporal control of Mas receptor activation. Inhalable liposomal and dry-powder formulations, cyclodextrin-based oral systems, biodegradable polymer carriers, and bioactive dendrimers address distinct pharmacokinetic barriers and offer complementary advantages depending on the intended route of administration and therapeutic objective (see also Table 3). From a translational perspective, lung-targeted delivery strategies, particularly inhaled nano- and micro-particulate formulations, represent the most rational near-term approach for Ang-(1–7) therapy in asthma and pulmonary fibrosis, as they enable high local drug concentrations while minimizing systemic exposure. Inhalable liposomal and dry-powder systems appear especially promising due to their compatibility with established pulmonary drug delivery technologies and favorable aerodynamic properties for deep lung deposition. Oral cyclodextrin-based formulations may serve as complementary options for long-term disease modulation or combination therapy in selected patient populations, particularly given the established clinical safety profile of HPβCD-containing formulations. In contrast, more complex multifunctional platforms such as PAMAM dendrimers and advanced polymeric nanocarriers, while mechanistically attractive due to their targeting and sustained-release capabilities, may face greater translational challenges related to large-scale manufacturing, long-term biocompatibility, regulatory complexity, and reproducibility of pharmacokinetic behavior. Similarly, implantable or continuous infusion systems provide important proof-of-concept for sustained Mas receptor activation but may be less practical for chronic outpatient management of pulmonary diseases (Figure 3). Future clinical development should prioritize disease-specific pharmacokinetic and pharmacodynamic profiling, inhalation safety, dose optimization, and biomarker-guided patient stratification, with rigorously designed early-phase clinical trials integrating advanced delivery platforms being essential to establish Ang-(1–7) as a disease-modifying therapy for chronic pulmonary diseases. Potential pharmacodynamic biomarkers for early-phase clinical trials may include circulating and bronchoalveolar inflammatory mediators such as IL-6, TNF-α, and TGF-β, alongside markers of oxidative stress, extracellular matrix remodeling, and ACE2/Ang-(1–7)/Mas axis activity. In pulmonary fibrosis, additional assessment of collagen turnover and fibrotic remodeling biomarkers may help evaluate biological response to therapy.

## 4 Human safety studies in clinical trials utilizing Ang-(1-7)

Since no clinical trials of Ang-(1-7) have been performed in asthma or PF patients, to evaluate the therapeutic feasibility of Ang-(1–7) for these conditions, it is essential to first contextualize its human safety profile based on evidence from other clinical trials conducted across diverse disease settings (see Table 4). Collectively, available clinical data demonstrate that Ang-(1–7) exhibits a consistently favorable safety and tolerability profile across a broad spectrum of patient populations, including inflammatory, cardiovascular, oncologic, and critical care cohorts. While dose-dependent adverse effects have been reported at higher systemic exposures, particularly with subcutaneous (SC) and intravenous (IV) administration, multiple studies have delineated clear exposure thresholds within which Ang-(1–7) remains both safe and biologically active (see also Table 4).

**TABLE 4:** Clinical safety studies of angiotensin-(1-7) and Mas receptor agonists.

#	Study	Study population	Demographics (n, setting)	Intervention (dose, route, frequency)	Outcome measures	Safety findings and AEs	Follow-up
1	Kono et al. (1986)	Human physiological interventional study comparing angiotensin peptides in healthy men and patients with Bartter’s syndrome. Single-center	N = 8; Group 1: 5 healthy men (age 26–33 years); Group 2: 3 patients with Bartter’s syndrome (age 15–33 years); Kyoto University, Japan	IV infusion of Ang-(1-7) at 18 nmol/kg/min for 15 min (A separate Ang-(1-6) infusion at 21 nmol/kg/min was also part of the study.)	Blood pressure response, PRA and plasma aldosterone	Healthy men: 5/5 (100%) had a significant, rapid pressor response (average BP increase +19/+15 mmHg at 2 min). Bartter’s syndrome patients: 0/3 (0%) had a significant change in BP or adverse effects	Acute single-day protocol; measurements before, during and immediately after the 15-min infusion. No longitudinal follow-up
2	Davie and McMurray (1999)	Human interventional study of the vascular effects of Ang-(1-7) and its interaction with bradykinin in heart-failure patients on ACE inhibitors. Single-center	N = 8; heart-failure patients (5M/3F, mean age 74 ± 4 years); all on ACE inhibitors; Western Infirmary, Glasgow, UK.	Local intra-brachial arterial infusion. (1) Ang-(1-7): 5, 50, 500, 5,000 and 50,000 pmol/min (6 min each). (2) Bradykinin: 3, 10 and 30 pmol/min (3 min each). (3) Co-infusion: bradykinin during ongoing Ang-(1-7) (50,000 pmol/min)	Vasodilation via forearm blood flow (venous occlusion plethysmography) and the Ang-(1-7)–bradykinin interaction	No adverse effects reported	Acute single-day protocol; measurements during and immediately after infusions. No longitudinal follow-up
3	Sasaki et al. (2001)	Human interventional study comparing the vascular effects of Ang-(1-7) in normotensive subjects and patients with essential hypertension. Single-center	N = 16; 8 normotensive (7M/1F, age 49.7 ± 11.2 years) and 8 hypertensive (6M/2F, age 54.6 ± 9.6 years); all Japanese; Hiroshima University, Japan	Intra-brachial arterial infusion of Ang-(1-7) at 10^−10^, 10^−9^ and 10^−8^ mol/min (5 min each). Mechanistic probe: co-infusion of the NO synthase inhibitor L-NMMA (8 μmol/min)	Forearm blood flow (FBF) by venous occlusion plethysmography; whether vasodilation is NO-mediated	No adverse effects related to Ang-(1-7). Minor discomfort from arterial catheterization (standard procedure risk)	Acute single-day protocol; measurements during infusion and a 30-min post-dose recovery period. No longitudinal follow-up
4	Plovsing et al. (2003)	Human interventional study of truncated angiotensin peptides during double RAAS blockade in healthy individuals. Single-center	N = 6; healthy male volunteers, age 22–28 years; all Danish; University of Southern Denmark and Odense University Hospital	Continuous IV infusion of Ang-(1-7) at 3 pmol/kg/min for 3 h. Comparators: Ang II, Ang III, Ang IV and placebo (glucose) at equimolar rates	Cardiovascular (BP), renal (sodium/potassium excretion, GFR) and hormonal (aldosterone, renin activity) effects	No AEs reported	Acute measurements during 5-h sessions (five 1-h urine collection periods); no long-term follow-up
5	Rodgers et al. (2006)	Phase I/II dose-escalation study in patients with newly diagnosed breast cancer receiving myelosuppressive chemotherapy	N = 21 randomized; 16 received Ang-(1-7) (mean age 51.4 years), 5 received filgrastim (mean age 54 years); all Caucasian	Ang-(1-7) at 2.5–100 μg/kg/day SC for 7 days pre-chemotherapy (Cycle 0) and ≥10 days post-chemotherapy (Cycles 1–3). Comparator: filgrastim 5 μg/kg/day SC. Chemotherapy: doxorubicin 60 mg/m^2^ + cyclophosphamide 600 mg/m^2^ every 21 days	Identify the optimal biological dose (OBD) of Ang-(1-7); assess hematologic recovery and toxicity	No DLTs or hypertension. Ang-(1-7) drug-related AEs: 10 events (4.3% of total), primarily musculoskeletal (8) and general (2). Serious AEs: febrile neutropenia (1 Ang-(1-7)), leukopenia (1 filgrastim), injection-site issue (1 filgrastim). Stomatitis lower with Ang-(1-7) (40%) vs. filgrastim (60%)	Cycle 0 and three 21-day cycles; hematology and vitals on days 1, 3, 5, 7, 9, 12, 15 and 21 of each cycle
6	Petty et al. (2009)	Phase I dose-escalating study in patients with advanced solid tumors refractory to standard therapy. Single-center, Wake Forest University Health Sciences	N = 18; 12 male (66.7%), 6 female (33.3%); mean age 61 years; 15 Caucasian (83.3%), 3 African American (16.7%); all non-Hispanic	SC Ang-(1-7) once daily for 5 consecutive days per 21-day cycle. Dose escalation by a 3 + 3 design from 100 μg/kg, with planned escalation to 200, 400, 700 and 1000 μg/kg	Establish the recommended Phase II dose; assess toxicities, PK, clinical activity (tumor response) and plasma biomarkers (PlGF, VEGF)	Non-serious AEs: muscle cramps (n = 3, 16.7%), fatigue (n = 2, 11.1%), musculoskeletal pain (n = 2, 11.1%), gingival regression, hypocalcemia, orthostasis and muscle weakness (each n = 1, 5.6%). Serious drug-related AEs: one DVT (Grade 3) at 400 μg/kg; one stroke (Grade 4) and one reversible cranial neuropathy (Grade 3) at 700 μg/kg. Generally well-tolerated	Tumor assessments every two cycles (42 days) until progression or unacceptable toxicity. Treatment 1 to 14 cycles (median 2)
7	Pham et al. (2013)	Phase 2, randomized, double-blind, placebo-controlled trial in patients with recurrent ovarian, fallopian tube or peritoneal carcinoma scheduled for gemcitabine/platinum. Multi-center, 6 US sites	N = 34; 100% female, median age 59 years (range 27–77); 64.7% (n = 22) ECOG status 0. Ethnicity not reported	SC TXA127 [Ang-(1-7)] at 100 μg/kg (n = 11) or 300 μg/kg (n = 13) vs. placebo (n = 10), daily on days 2–6 and 9–15 of each 21-day cycle for up to 6 cycles, concurrent with chemotherapy	Reduction in chemotherapy-induced thrombocytopenia (Grade 3–4), platelet support (maximal % increase) and maintenance of scheduled dose intensity	Drug-related: one patient (9.1%) in the 100 μg/kg group developed thrombocytosis (platelets 824 × 10^9^/L), leading to discontinuation (resolved spontaneously). Non-drug-related: comparable nausea (67.6%), fatigue (61.8%), constipation (58.8%), neutropenia (55.9%), anemia (32.4%) and headache (50.0%), attributed to chemotherapy. No systemic hemodynamic changes or severe toxicities linked to Ang-(1-7)	Up to 6 cycles (21 days each; max 126 days). Hematologic parameters weekly; no post-treatment follow-up beyond the final cycle
8	Van Twist et al. (2013)	Randomized, parallel-group physiological interventional study in essential hypertensive patients undergoing renal angiography. Maastricht University Medical Centre, Netherlands	N = 57; 35 male, 22 female, 100% White, mean age 54.5 years; difficult-to-treat hypertension (BP above goal despite ≥3 full-dose antihypertensives)	7-day low-sodium (<55 mmol/day; n = 32) or high-sodium (>200 mmol/day; n = 25) diet. Within each group, intrarenal Ang-(1-7) alone (0.27, 0.9, 2.7 ng/kg/min; 5 min each) or with continuous Ang II co-infusion (0.3 ng/kg/min)	Renal blood flow response (mean renal blood flow via^130^Xenon washout)	No AEs reported	Acute phase only; measurements during infusion and angiography. No long-term follow-up
9	Becker et al. (2018)	Randomized, double-blind, placebo-controlled, parallel-group trial in healthy adults undergoing eccentric overload exercise in Brazil	N = 25; 21 males (84.0%) and 4 females (16.0%), mean age 25 years. Ethnicity not reported. No comorbidities	Fixed-dose oral HPβCD/Ang-(1-7) (1.75 mg) once daily for 7 days, initiated 3 h pre-exercise, vs. vehicle placebo (HPβCD capsules); no dose escalation	Post-exercise muscle soreness (visual analogue scale), CK levels, maximal isometric strength and plasma cytokines (TNF-α, IL-6, IL-10)	No AEs reported	Assessments at baseline, immediately post-exercise and 24, 48, 72 and 168 h post-exercise
10	Wagener et al. (2022)	Phase 2 randomized, double-blind, placebo-controlled pilot study in hospitalized patients with severe COVID-19 requiring supplemental oxygen. Single-center	N = 20; median age 57 (TXA-127) and 55 (placebo) y; 13 males (65%), 7 females (35%); Columbia University Irving Medical Center, New York, USA.	IV TXA-127 at 0.5 mg/kg daily vs. placebo for a planned 10 days (median actual 4.5 days due to discharge or deterioration)	Incidence of AKI and respiratory failure requiring mechanical ventilation	Most common AEs: cough, headache, chest discomfort (no significant between-group difference). 4 required mechanical ventilation (3 TXA-127, 1 placebo). 3 deaths (2 TXA-127, 1 placebo). 4 developed AKI (3 TXA-127, 1 placebo). No SAEs attributed to TXA-127	Followed during hospitalization; median drug administration 4.5 days; median hospital stay 7–11 days
11	Robbins et al. (2023)	Double-blind, randomized, placebo-controlled trial in hospitalized patients with severe COVID-19 and coagulopathy	N = 21; median age 72 (control) and 70.5 (TRV027) y; mixed ethnicity; all received anticoagulants; single-center, UK.	TRV027 at 12 mg/h via continuous IV infusion vs. 0.9% sodium chloride (placebo) for ≤7 days or until discharge	D-dimer change at Day 3; secondary coagulation markers (fibrinogen, aPTT, INR), hematological/biochemical parameters and physiological observations	4/10 in the TRV027 arm had SAEs (2 deaths from COVID-19 pneumonia, 1 pulmonary embolism, 1 steroid-induced confusion). 3/11 in placebo had SAEs (1 death, 1 bacterial sepsis, 1 pulmonary embolism). All SAEs deemed unrelated to study drug. Non-serious AEs infrequent	Assessments at baseline (Day 1), Day 3 (primary endpoint), Day 5 and Day 8
12	Martins et al. (2024)	Phase I, open-label, investigator-initiated trial in ICU COVID-19 patients with severe pneumonia. Multicenter (2 ICUs in Brazil)	N = 28; mean age 55.8 years; 78.6% male; White (35.7%), Brown (50.0%), Black (7.1%). Comorbidities: hypertension (46.4%), diabetes (35.7%), obesity (39.2%)	Continuous IV infusion of Ang-(1-7) at 10 μg/kg/day for up to 7 days	Incidence of SAEs; secondary: organ-failure-free days by day 28, ICU length of stay, mortality, ventilator-free days and vasopressor need	Generally well-tolerated. One patient (3.6%) developed transient sinus bradycardia (resolved after dose reduction). 5 required vasopressors (4 low-dose, 1 high-dose due to septic shock). 6 deaths (21.4%), all deemed unrelated to study drug. No drug-induced hypotension, fever or thrombotic complications	Clinical outcomes and safety monitored for 28 days after inclusion
12	Silva De Moura et al. (2024)	Double-blind, randomized crossover trial in recreational mountain bike athletes. Single-center	N = 21; male, mean ages 33.5 and 35.5 years, ≥3 years competitive experience, training ≥5 days/week; Federal University of Ouro Preto, Brazil	Oral HPβCD-Ang-(1-7) at 0.8 mg/day vs. HPβCD-placebo, capsules taken 3 h before exercise, for 7 days in a crossover design with a washout period	Physical performance (time-trial time, max/mean sprint power), fatigue parameters (lactate, EMG), cardiovascular parameters (BP, HR) and recovery biomarkers (glucose, nitrite, NEFAs)	No adverse effects reported. One participant withdrew due to unrelated scheduling conflicts	Assessments immediately post-exercise and after the 7-day intervention period

Studies are ordered chronologically by year of publication. TXA-127, and TRV027 are clinical formulations/agonists acting on the Ang-(1-7)/Mas axis. All abbreviations are defined in Supplementary Table S1.

SC administration at doses of 100 μg/kg/day has demonstrated robust therapeutic efficacy, most notably in oncology patients, where Ang-(1–7) significantly reduced chemotherapy-induced thrombocytopenia (Pham et al., 2013). Although attribution of adverse events was complicated by overlapping toxicities from concomitant chemotherapy, higher doses exceeding 300 μg/kg/day appeared to correlate with an increased incidence of peripheral neuropathy and thrombotic complications (Petty et al., 2009; Pham et al., 2013). These observations underscore the importance of careful dose titration and patient-specific risk stratification when employing systemic Ang-(1–7) therapy.

Continuous IV infusion has similarly demonstrated favorable tolerability at doses up to 10 μg/kg/day. In critically ill COVID-19 patients, this regimen was associated with improvements in oxygenation and multi-organ function without evidence of renal, cardiovascular, or hemodynamic toxicity (Robbins et al., 2023; Martins et al., 2024). Importantly, no significant differences in D-dimer levels or thromboembolic events were observed compared with placebo, reinforcing the compound’s reassuring coagulation safety profile even in high-risk inflammatory states. Notably, a single episode of transient bradycardia was reported, suggesting that this dose may approximate the upper physiological safety threshold in acutely ill populations.

In contrast, a 15-min IV infusion of Ang-(1–7) at 18 nmol/kg/min in healthy volunteers elicited significant, though modest, pressor responses, averaging up to +20/+17 mmHg (Kono et al., 1986). These findings indicate that even controlled IV infusion can elicit measurable activation of vascular angiotensin receptors. By extension, bolus IV administration, delivering an equivalent or greater peptide dose over a markedly shorter timeframe, would be expected to produce a more abrupt and pronounced hemodynamic response, with a heightened risk of acute hypertension. Accordingly, bolus IV dosing of Ang-(1–7) should be avoided and, if ever considered, restricted to closely monitored inpatient settings.

Oral formulations of Ang-(1–7), particularly hydroxypropyl β-cyclodextrin (HPβCD)-conjugated preparations, have demonstrated excellent tolerability and anti-inflammatory potential. In clinical studies, a total capsule dose of 1.75 mg was used without reported adverse effects (Becker et al., 2018), while a separate study administered 0.8 mg of Ang-(1–7) in 2 mg capsules to healthy athletes, similarly demonstrating a favorable safety profile (Silva De Moura et al., 2024). Moreover, this oral formulation was associated with reductions in TNF-α levels and preservation of post-exercise glycemic stability, suggesting that an oral HPβCD–Ang-(1–7) dose of approximately 0.8 mg/day may represent a practical and safe therapeutic strategy for chronic inflammatory conditions such as asthma and PF.

Additional insights into the hemodynamic safety of Ang-(1–7) emerge from studies employing intra-arterial infusion. In normotensive and hypertensive subjects receiving 50 pmol/kg/min via the brachial artery, Ang-(1–7) induced localized vasodilation without provoking systemic hypotension or reflex tachycardia (Sasaki et al., 2001). Complementary investigations within the renal vasculature demonstrated that Ang-(1–7) promoted renal vasodilation in hypertensive patients; however, this effect was attenuated under low-sodium conditions or during co-infusion with Angiotensin II, highlighting the modulatory influence of baseline renin–angiotensin system activity (Van Twist et al., 2013). These findings emphasize the context-dependent vascular actions of Ang-(1–7) and the necessity of accounting for underlying physiological states when considering its therapeutic application.

Despite these encouraging safety data, several limitations remain. Notably, no clinical trials to date have evaluated Ang-(1–7) specifically in asthma or PF, substantially limiting disease-specific conclusions. Existing studies predominantly involve oncologic, cardiovascular, renal, or critically ill COVID-19 populations, with sparse data on dose–response relationships, long-term exposure, or pulmonary-specific endpoints. Furthermore, safety outcomes have been inconsistently reported, and study heterogeneity in dosing strategies, administration routes, and clinical endpoints constrains cross-study comparability. Taken together, current human data strongly support the overall safety and tolerability of Ang-(1–7) across multiple clinical contexts, while simultaneously underscoring the urgent need for rigorously designed, respiratory disease–focused clinical trials. Such studies are essential to define optimal dosing, delivery strategies, and long-term safety profiles in asthma and fibrotic lung disease to facilitate informed clinical translation.

## 5 Conclusion

In summary, converging preclinical and some early clinical evidence supports Ang-(1–7) as a compelling therapeutic candidate for asthma and PF. The bulk of the evidence comes from experimental models of asthma where Ang-(1–7) consistently exerts anti-inflammatory, anti-fibrotic, and anti-remodeling effects through suppression of Th2-driven cytokine signaling, inhibition of EGFR transactivation, attenuation of oxidative stress, and restoration of autophagic homeostasis. These coordinated actions translate into improved lung function, reduced AHR, and marked attenuation of structural airway remodeling. Likewise, in preclinical models of PF, Ang-(1–7) limits fibrotic progression by modulating the NOX4–Nrf2 redox axis, inhibiting profibrotic TGF-β/SMAD and Hedgehog signaling pathways, and restoring redox balance, thereby reducing collagen deposition and preserving alveolar architecture. It should be noted that several of the preclinical studies rely on preventative administration paradigms rather than treating established disease. Thus, the ability of Ang-(1-7) to reverse established pulmonary fibrosis and act as a true disease-modifying therapy remains a key translational gap that warrants further investigation.

Importantly, clinical studies conducted across diverse disease contexts have demonstrated a favorable safety and tolerability profile for Ang-(1–7). Notably, orally administered HPβCD-formulated Ang-(1–7) at doses of approximately 0.8 mg/day exhibited anti-inflammatory activity without significant adverse effects, supporting the feasibility of long-term administration in chronic disease settings. Nevertheless, localized pulmonary delivery may offer distinct therapeutic advantages by enabling direct targeting of diseased lung tissue, reducing systemic exposure, and lowering required doses. In this regard, advanced nano-enabled delivery platforms represent a particularly promising strategy to overcome the intrinsic pharmacokinetic limitations of Ang-(1–7).

Despite these encouraging findings, the absence of disease-specific clinical trials evaluating Ang-(1–7), either alone or in optimized nano-formulations, constitutes a critical translational gap. Addressing this gap will require rigorously designed, adequately powered clinical trials in asthma and PF to define therapeutic efficacy, optimal dosing strategies, long-term safety, and mechanistic biomarkers of response. Collectively, such efforts are essential to establish Ang-(1–7) as a *bona fide* disease-modifying therapy for chronic pulmonary diseases.

## References

[B1] Aghazadeh-Habashi A. Khajehpour S. (2021). Improved pharmacokinetics and bone tissue accumulation of Angiotensin-(1–7) peptide through bisphosphonate conjugation. Amino Acids 53, 653–664. 10.1007/s00726-021-02972-2 33791863

[B2] Akhtar S. Benter I. (2007). Toxicogenomics of non-viral drug delivery systems for RNAi: potential impact on siRNA-mediated gene silencing activity and specificity. Adv. Drug Deliv. Rev. 59, 164–182. 10.1016/j.addr.2007.03.010 17481774

[B3] Akhtar S. Yousif M. H. Dhaunsi G. S. Chandrasekhar B. Al‐Farsi O. Benter I. F. (2012). Angiotensin‐(1‐7) inhibits epidermal growth factor receptor transactivation *via* a mas receptor‐dependent pathway. Br. J Pharmacol. 165, 1390–1400. 10.1111/j.1476-5381.2011.01613.x 21806601 PMC3372724

[B4] Akhtar S. Chandrasekhar B. Attur S. Yousif M. H. M. Benter I. F. (2013). On the nanotoxicity of PAMAM dendrimers: superfect® stimulates the EGFR–ERK1/2 signal transduction pathway *via* an oxidative stress-dependent mechanism in HEK 293 cells. Int. J. Pharm. 448, 239–246. 10.1016/j.ijpharm.2013.03.039 23538097

[B5] Akhtar S. Al-Zaid B. El-Hashim A. Z. Chandrasekhar B. Attur S. Yousif M. H. M. (2015). Cationic polyamidoamine dendrimers as modulators of EGFR signaling *in vitro* and *in vivo* . PLoS ONE 10, e0132215. 10.1371/journal.pone.0132215 26167903 PMC4500564

[B6] Akhtar S. Al-Zaid B. El-Hashim A. Z. Chandrasekhar B. Attur S. Benter I. F. (2016a). Impact of PAMAM delivery systems on signal transduction pathways *in vivo:* modulation of ERK1/2 and p38 MAP kinase signaling in the normal and diabetic kidney. Int. J. Pharm. 514, 353–363. 10.1016/j.ijpharm.2016.03.039 27032566

[B7] Akhtar S. El-Hashim A. Z. Chandrasekhar B. Attur S. Benter I. F. (2016b). Naked polyamidoamine polymers intrinsically inhibit angiotensin II-Mediated EGFR and ErbB2 transactivation in a dendrimer Generation- and surface chemistry-dependent manner. Mol. Pharm. 13, 1575–1586. 10.1021/acs.molpharmaceut.6b00045 26985693

[B8] Akhtar S. Chandrasekhar B. Yousif M. H. Renno W. Benter I. F. El-Hashim A. Z. (2019). Chronic administration of nano-sized PAMAM dendrimers *in vivo* inhibits EGFR-ERK1/2-ROCK signaling pathway and attenuates diabetes-induced vascular remodeling and dysfunction. Nanomedicine Nanotechnol. Biol. Med. 18, 78–89. 10.1016/j.nano.2019.02.012 30844576

[B9] Akhtar S. Benter I. F. Danjuma M. I. Doi S. A. R. Hasan S. S. Habib A. M. (2020). Pharmacotherapy in COVID-19 patients: a review of ACE2-raising drugs and their clinical safety. J. Drug Target 28 (7-8), 683–699. 10.1080/1061186X.2020.1797754 32700580

[B10] Akhtar S. Babiker F. Akhtar U. A. Benter I. F. (2022). Mitigating cardiotoxicity of dendrimers: angiotensin-(1-7) *via* its mas receptor ameliorates PAMAM-induced cardiac dysfunction in the isolated mammalian heart. Pharmaceutics 14, 2673. 10.3390/pharmaceutics14122673 36559167 PMC9781033

[B11] Akhtar S. Babiker F. Al-Kouh A. F Benter I. (2025). The cardiac toxicity of PAMAM dendrimer drug delivery systems can be attenuated with the adjunct use of cardioprotective agents. Biomol. Biomed. 25, 914–924. 10.17305/bb.2024.10735 39319862 PMC11959388

[B12] Al-Maghrebi M. Benter I. F. Diz D. I. (2009). Endogenous angiotensin-(1-7) reduces cardiac ischemia-induced dysfunction in diabetic hypertensive rats. Pharmacol. Res. 59, 263–268. 10.1016/j.phrs.2008.12.008 19166939 PMC2682227

[B13] Alabsi W. Acosta M. F. Al-Obeidi F. A. Hay M. Polt R. Mansour H. M. (2021). Synthesis, physicochemical characterization, *in vitro* 2D/3D human cell culture, and *in vitro* aerosol dispersion performance of advanced spray dried and Co-Spray dried angiotensin (1—7) peptide and PNA5 with trehalose as microparticles/nanoparticles for targeted respiratory delivery as dry powder inhalers. Pharmaceutics 13, 1278. 10.3390/pharmaceutics13081278 34452239 PMC8398878

[B14] Alamos-Musre S. Beltrán-Chacana D. Moyano J. Márquez-Miranda V. Duarte Y. Miranda-Rojas S. (2025). From structure to function: the promise of PAMAM dendrimers in biomedical applications. Pharmaceutics 17, 927. 10.3390/pharmaceutics17070927 40733135 PMC12299782

[B15] Babiker F. Benter I. F. Akhtar S. (2020). Nanotoxicology of dendrimers in the mammalian heart: *ex vivo* and *in vivo* administration of G6 PAMAM nanoparticles impairs recovery of cardiac function following ischemia-reperfusion injury. IJN 15, 4393–4405. 10.2147/IJN.S255202 32606684 PMC7310973

[B16] Bader M. Steckelings U. M. Alenina N. Santos R. A. S. Ferrario C. M. (2024). Alternative renin-angiotensin system. Hypertension 81, 964–976. 10.1161/HYPERTENSIONAHA.123.21364 38362781 PMC11023806

[B17] Balakumar P. Jagadeesh G. (2014). A century old renin–angiotensin system still grows with endless possibilities: AT1 receptor signaling cascades in cardiovascular physiopathology. Cell. Signal. 26, 2147–2160. 10.1016/j.cellsig.2014.06.011 25007996

[B18] Banno A. Reddy A. T. Lakshmi S. P. Reddy R. C. (2020). Bidirectional interaction of airway epithelial remodeling and inflammation in asthma. Clin. Sci. 134, 1063–1079. 10.1042/CS20191309 32369100

[B19] Becker L. Totou N. Moura S. Kangussu L. Millán R. Campagnole-Santos M. (2018). Eccentric overload muscle damage is attenuated by a novel Angiotensin- (1-7) treatment. Int. J. Sports Med. 39, 743–748. 10.1055/a-0633-8892 29940668

[B20] Benter I. F. Diz D. I. Ferrario C. M. (1993). Cardiovascular actions of angiotensin(1–7). Peptides 14, 679–684. 10.1016/0196-9781(93)90097-Z 8234010

[B21] Benter I. F. Diz D. I. Ferrario C. M. (1995a). Pressor and reflex sensitivity is altered in spontaneously hypertensive rats treated with Angiotensin-(1-7). Hypertension 26, 1138–1144. 10.1161/01.HYP.26.6.1138 7498984

[B22] Benter I. F. Ferrario C. M. Morris M. Diz D. I. (1995b). Antihypertensive actions of angiotensin-(1-7) in spontaneously hypertensive rats. Am. J. Physiology-Heart Circulatory Physiology 269, H313–H319. 10.1152/ajpheart.1995.269.1.H313 7631863

[B23] Benter I. F. Yousif M. H. M. Anim J. T. Cojocel C. Diz D. I. (2006). Angiotensin-(1–7) prevents development of severe hypertension and end-organ damage in spontaneously hypertensive rats treated with l -NAME. Am. J. Physiology-Heart Circulatory Physiology 290, H684–H691. 10.1152/ajpheart.00632.2005 16403946

[B24] Benter I. F. Yousif M. H. M. Cojocel C. Al-Maghrebi M. Diz D. I. (2007). Angiotensin-(1–7) prevents diabetes-induced cardiovascular dysfunction. Am. J. Physiology-Heart Circulatory Physiology 292, H666–H672. 10.1152/ajpheart.00372.2006 17213482

[B25] Benter I. F. Yousif M. H. M. Dhaunsi G. S. Kaur J. Chappell M. C. Diz D. I. (2008). Angiotensin-(1–7) prevents activation of NADPH oxidase and renal vascular dysfunction in diabetic hypertensive rats. Am. J. Nephrol. 28, 25–33. 10.1159/000108758 17890855

[B26] Bernstein K. E. Ong F. S. Blackwell W.-L. B. Shah K. H. Giani J. F. Gonzalez-Villalobos R. A. (2013). A modern understanding of the traditional and nontraditional biological functions of angiotensin-converting enzyme. Pharmacol. Rev. 65, 1–46. 10.1124/pr.112.006809 23257181 PMC3565918

[B27] Bonsignore C. L. (1998). Inhaled tobramycin (TOBI). Pediatr. Nurs. 24, 258–259. 9987427

[B28] Cao Y. Liu Y. Shang J. Yuan Z. Ping F. Yao S. (2019). Ang-(1-7) treatment attenuates lipopolysaccharide-induced early pulmonary fibrosis. Lab. Investig. 99, 1770–1783. 10.1038/s41374-019-0289-7 31278346

[B29] Chauhan A. S. Diwan P. V. Jain N. K. Tomalia D. A. (2009). Unexpected *in vivo* anti-inflammatory activity observed for simple, surface functionalized poly(amidoamine) dendrimers. Biomacromolecules 10, 1195–1202. 10.1021/bm9000298 19348417

[B30] Chen Q. Yang Y. Huang Y. Pan C. Liu L. Qiu H. (2013). Angiotensin-(1-7) attenuates lung fibrosis by way of mas receptor in acute lung injury. J. Surg. Res. 185, 740–747. 10.1016/j.jss.2013.06.052 23890397

[B31] Chi L. A. Asgharpour S. Correa-Basurto J. Bandala C. R. Martínez-Archundia M. (2022). Unveiling the G4-PAMAM capacity to bind and protect Ang-(1-7) bioactive peptide by molecular dynamics simulations. J. Comput. Aided Mol. Des. 36, 653–675. 10.1007/s10822-022-00470-5 35934747 PMC9358120

[B32] Chis A. A. Dobrea C. Morgovan C. Arseniu A. M. Rus L. L. Butuca A. (2020). Applications and limitations of dendrimers in biomedicine. Molecules 25, 3982. 10.3390/molecules25173982 32882920 PMC7504821

[B33] Cuoco A. Eriksen J. B. Luppi B. Brandl M. Bauer-Brandl A. (2023). When interactions between bile salts and Cyclodextrin cause a negative food effect: dynamic dissolution/permeation studies with itraconazole (sporanox®) and biomimetic media. J. Pharm. Sci. 112, 1372–1378. 10.1016/j.xphs.2022.12.010 36539063

[B34] Da Silva W. Carvalho Costa P. A. Scalzo Júnior S. R. Ferreira H. Prazeres P. H. Campos C. (2024). Ionizable lipid nanoparticle-mediated TRAIL mRNA delivery in the tumor microenvironment to inhibit Colon cancer progression. IJN 19, 2655–2673. 10.2147/IJN.S452896 38500680 PMC10946446

[B35] Daniell H. Wakade G. Nair S. K. Singh R. Emanuel S. A. Brock B. (2024). Evaluation of biologics ACE2/Ang(1–7) encapsulated in plant cells for FDA approval: safety and toxicology studies. Pharmaceutics 17, 12. 10.3390/pharmaceutics17010012 39861664 PMC11768411

[B36] Davie A. P. McMurray J. J. V. (1999). Effect of Angiotensin-(1-7) and bradykinin in patients with heart failure treated with an ACE inhibitor. Hypertension 34, 457–460. 10.1161/01.HYP.34.3.457 10489393

[B37] Delaitre C. Boisbrun M. Lecat S. Dupuis F. (2021). Targeting the angiotensin II type 1 receptor in cerebrovascular diseases: biased signaling raises new hopes. IJMS 22, 6738. 10.3390/ijms22136738 34201646 PMC8269339

[B38] Dhaunsi G. S. Yousif M. H. M. Akhtar S. Chappell M. C. Diz D. I. Benter I. F. (2010). Angiotensin-(1–7) prevents diabetes-induced attenuation in PPAR-γ and catalase activities. Eur. J. Pharmacol. 638, 108–114. 10.1016/j.ejphar.2010.04.030 20447391 PMC2907528

[B39] Donoghue M. Hsieh F. Baronas E. Godbout K. Gosselin M. Stagliano N. (2000). A novel angiotensin-converting enzyme-related carboxypeptidase (ACE2) converts angiotensin I to angiotensin 1-9. Circ. Res. 87, E1–E9. 10.1161/01.res.87.5.e1 10969042

[B153] El-Hashim A. Z. Khajah M. A. Renno W. M. Babyson R. S. Uddin M. Benter I. F. (2017). Src-dependent EGFR transactivation regulates lung inflammation via downstream signaling involving ERK1/2, PI3Kδ/Akt and NFκB induction in a murine asthma model. Sci. Rep. 7 (1), 9919. 10.1038/s41598-017-09349-0 28855674 PMC5577320

[B40] El-Hashim A. Z. Khajah M. A. Babyson R. S. Renno W. M. Ezeamuzie C. I. Benter I. F. (2019). Ang-(1-7)/MAS1 receptor axis inhibits allergic airway inflammation *via* blockade of src-mediated EGFR transactivation in a murine model of asthma. PLoS ONE 14, e0224163. 10.1371/journal.pone.0224163 31675376 PMC6824568

[B41] El‐Hashim A. Z. Renno W. M. Raghupathy R. Abduo H. T. Akhtar S. Benter I. F. (2012). Angiotensin‐(1–7) inhibits allergic inflammation, *via* the MAS1 receptor, through suppression of ERK1/2‐ and NF‐κB‐dependent pathways. Br. J Pharmacol. 166, 1964–1976. 10.1111/j.1476-5381.2012.01905.x 22339213 PMC3402818

[B42] Encinas-Basurto D. Konhilas J. P. Polt R. Hay M. Mansour H. M. (2022). Glycosylated Ang-(1-7) MasR agonist peptide poly Lactic-co-Glycolic acid (PLGA) nanoparticles and microparticles in cognitive impairment: design, particle preparation, physicochemical characterization, and *in vitro* release. Pharmaceutics 14, 587. 10.3390/pharmaceutics14030587 35335963 PMC8954495

[B43] Fang J. Sawa T. Maeda H. (2004). “Factors and mechanism of ‘EPR’ effect and the enhanced antitumor effects of macromolecular drugs including SMANCS,” in Polymer Drugs in the Clinical Stage. Editors Maeda H. Kabanov A. Kataoka K. Okano T. (Boston: Kluwer Academic Publishers), 29–49. 10.1007/0-306-47932-X_2 12675206

[B44] Figueiredo V. P. Barbosa M. A. De Castro U. G. M. Zacarias A. C. Bezerra F. S. De Sá R. G. (2019). Antioxidant effects of oral Ang-(1-7) restore insulin pathway and RAS components ameliorating cardiometabolic disturbances in rats. Oxidative Med. Cell. Longev. 2019, 1–10. 10.1155/2019/5868935 31396301 PMC6664692

[B45] Finkelman F. D. Hogan S. P. Hershey G. K. K. Rothenberg M. E. Wills-Karp M. (2010). Importance of cytokines in murine allergic airway disease and human asthma. J. Immunol. 184, 1663–1674. 10.4049/jimmunol.0902185 20130218 PMC4122090

[B46] Fleming L. W. Fleming J. W. Davis C. S. (2015). Afrezza: an inhaled approach to insulin delivery. J. Am. Assoc. Nurse Pract. 27, 597–601. 10.1002/2327-6924.12247 25764151

[B47] Fraga-Silva R. A. Costa-Fraga F. P. De Sousa F. B. Alenina N. Bader M. Sinisterra R. D. (2011). An orally active formulation of angiotensin-(1-7) produces an antithrombotic effect. Clinics 66, 837–841. 10.1590/S1807-59322011000500021 21789389 PMC3109384

[B48] Gallagher P. E. Arter A. L. Deng G. Tallant E. A. (2014). Angiotensin-(1-7): a peptide hormone with anti-cancer activity. Curr. Med. Chem. 21, 2417–2423. 10.2174/0929867321666140205133357 24524765

[B49] Gan P. X. L. Liao W. Linke K. M. Mei D. Wu X. D. Wong W. S. F. (2023). “Targeting the renin angiotensin system for respiratory diseases,” in Advances in Pharmacology (Elsevier), 111–144. 10.1016/bs.apha.2023.02.002 37524485

[B50] Gao X. Xu H. Zhang B. Tao T. Liu Y. Xu D. (2019). Interaction of *N* ‐acetyl‐seryl‐aspartyl‐lysyl‐proline with the angiotensin‐converting enzyme 2–angiotensin‐(1–7)–Mas axis attenuates pulmonary fibrosis in silicotic rats. Exp. Physiol. 104, 1562–1574. 10.1113/EP087515 31290182

[B51] Gava E. Samad-Zadeh A. Zimpelmann J. Bahramifarid N. Kitten G. T. Santos R. A. (2009). Angiotensin-(1-7) activates a tyrosine phosphatase and inhibits glucose-induced signalling in proximal tubular cells. Nephrol. Dial. Transplant. 24, 1766–1773. 10.1093/ndt/gfn736 19144997 PMC2684752

[B52] Gironacci M. M. Adamo H. P. Corradi G. Santos R. A. Ortiz P. Carretero O. A. (2011). Angiotensin (1-7) induces MAS receptor internalization. Hypertension 58, 176–181. 10.1161/HYPERTENSIONAHA.111.173344 21670420 PMC3141282

[B53] Gonçalves S. C. A. Gregório J. F. Ferraz K. S. Magalhães G. S. Zebral S. A. Fontes M. A. P. (2025). Oral or intranasal angiotensin-(1-7) improves anxiety and depression-like behaviors in mice subjected to allergic pulmonary inflammation. Behav. Brain Res. 494, 115744. 10.1016/j.bbr.2025.115744 40684808

[B54] Gregório J. F. Magalhães G. S. Rodrigues-Machado M. G. Gonzaga K. E. R. Motta-Santos D. Cassini-Vieira P. (2021). Angiotensin-(1-7)/Mas receptor modulates anti-inflammatory effects of exercise training in a model of chronic allergic lung inflammation. Life Sci. 282, 119792. 10.1016/j.lfs.2021.119792 34229006

[B55] Hay M. Polt R. Heien M. L. Vanderah T. W. Largent-Milnes T. M. Rodgers K. (2019). A novel Angiotensin-(1-7) glycosylated mas receptor agonist for treating vascular cognitive impairment and inflammation-related memory dysfunction. J. Pharmacol. Exp. Ther. 369, 9–25. 10.1124/jpet.118.254854 30709867 PMC6413771

[B56] He X. Zou J. Zhang W. Ma Y. He W. (2025). Pulmonary inhaled drug delivery systems: from bench to bedside. J. Control Release 388 (Pt 1), 114296. 10.1016/j.jconrel.2025.114296 41067598

[B57] Heinemann L. Baughman R. Boss A. Hompesch M. (2017). Pharmacokinetic and pharmacodynamic properties of a novel inhaled insulin. J. Diabetes Sci. Technol. 11, 148–156. 10.1177/1932296816658055 27378794 PMC5375067

[B58] Hirsch I. B. Beck R. W. Marak M. C. Calhoun P. Mottalib A. Salhin A. (2024). A randomized comparison of postprandial glucose excursion using inhaled insulin *versus* rapid-acting analog insulin in adults with type 1 diabetes using multiple daily injections of insulin or automated insulin delivery. Diabetes Care 47, 1682–1687. 10.2337/dc24-0838 39042575 PMC11362108

[B59] Hollins A. J. Omidi Y. Benter I. F. Akhtar S. (2007). Toxicogenomics of drug delivery systems: exploiting delivery system-induced changes in target gene expression to enhance siRNA activity. J. Drug Target. 15, 83–88. 10.1080/10611860601151860 17365277

[B60] Hoyer-Kimura C. Konhilas J. P. Mansour H. M. Polt R. Doyle K. P. Billheimer D. (2021). Neurofilament light: a possible prognostic biomarker for treatment of vascular contributions to cognitive impairment and dementia. J. Neuroinflammation 18, 236. 10.1186/s12974-021-02281-1 34654436 PMC8520282

[B61] Huang X. Yu W. Wei A. Wang X. Chen S. (2025). Beyond tumors: the pivotal role of TRIM proteins in chronic non-tumor lung diseases. JIR 18, 1899–1910. 10.2147/JIR.S499029 PMC11812559 39935527

[B62] Ishmael L. Casale T. Cardet J. C. (2024). Molecular pathways and potential therapeutic targets of refractory asthma. Biology 13, 583. 10.3390/biology13080583 39194521 PMC11351281

[B63] Jambhekar S. S. Breen P. (2016). Cyclodextrins in pharmaceutical formulations I: structure and physicochemical properties, formation of complexes, and types of complex. Drug Discov. Today 21, 356–362. 10.1016/j.drudis.2015.11.017 26686054

[B64] Jayawardena T. U. Nagahawatta D. P. Grant M. B. Chappell M. C. Oudit G. Y. (2026). Therapeutic opportunities targeting the ACE2/Ang-(1-7)/MasR pathway in cardiometabolic disease. Curr. Hypertens. Rep. 28, 18. 10.1007/s11906-026-01364-9 41838282

[B65] Khalfaoui L. Pabelick C. M. (2023). Airway smooth muscle in contractility and remodeling of asthma: potential drug target mechanisms. Expert Opin. Ther. Targets 27, 19–29. 10.1080/14728222.2023.2177533 36744401 PMC10208413

[B66] Kheraldine H. Rachid O. Habib A. M. Al Moustafa A.-E. Benter I. F. Akhtar S. (2021). Emerging innate biological properties of nano-drug delivery systems: a focus on PAMAM dendrimers and their clinical potential. Adv. Drug Deliv. Rev. 178, 113908. 10.1016/j.addr.2021.113908 34390777

[B67] Kim K.-S. Abraham D. Williams B. Violin J. D. Mao L. Rockman H. A. (2012). β-Arrestin-biased AT1R stimulation promotes cell survival during acute cardiac injury. Am. J. Physiology-Heart Circulatory Physiology 303, H1001–H1010. 10.1152/ajpheart.00475.2012 22886417 PMC3469643

[B68] Kluskens L. D. Nelemans S. A. Rink R. De Vries L. Meter-Arkema A. Wang Y. (2009). Angiotensin-(1-7) with thioether bridge: an angiotensin-converting enzyme-resistant, potent Angiotensin-(1-7) analog. J. Pharmacol. Exp. Ther. 328, 849–854. 10.1124/jpet.108.146431 19038778

[B69] Kolb P. Upagupta C. Vierhout M. Ayaub E. Bellaye P. S. Gauldie J. (2020). The importance of interventional timing in the bleomycin model of pulmonary fibrosis. Eur. Respir. J. 55, 1901105. 10.1183/13993003.01105-2019 32165401

[B70] Kono T. Taniguchi A. Imura H. Oseko F. Khosla M. C. (1986). Biological activities of angiotensin II-(1–6)-hexapeptide and angiotensin II-(1–7)-heptapeptide in man. Life Sci. 38, 1515–1519. 10.1016/0024-3205(86)90565-5 3702589

[B71] Koudstaal T. Funke-Chambour M. Kreuter M. Molyneaux P. L. Wijsenbeek M. S. (2023). Pulmonary fibrosis: from pathogenesis to clinical decision-making. Trends Mol. Med. 29, 1076–1087. 10.1016/j.molmed.2023.08.010 37716906

[B72] Kuriakose J. Montezano A. C. Touyz R. M. (2021). ACE2/Ang-(1-7)/Mas1 axis and the vascular system: vasoprotection to COVID-19-associated vascular disease. Clin. Sci. 135, 387–407. 10.1042/CS20200480 PMC7846970 33511992

[B73] Lee D.-K. Kim G. Maruthupandy M. Lee K. Cho W.-S. (2024). Multimodal pulmonary clearance kinetics of carbon Black nanoparticles deposited in the lungs of rats: the role of alveolar macrophages. Part Fibre Toxicol. 21, 32. 10.1186/s12989-024-00591-9 39135079 PMC11318259

[B74] Li R. Jia Y. Kong X. Nie Y. Deng Y. Liu Y. (2022). Novel drug delivery systems and disease models for pulmonary fibrosis. J. Control Release 348, 95–114. 10.1016/j.jconrel.2022.05.039 35636615

[B75] Li L. Zhao L. Li M. Tao Y. Sabri A. H. B. Moreno-Castellanos N. (2025). Schizophrenia treatment based on sustained release of risperidone from Poly(lactic-*co* -glycolic) acid implantable microarray patch. ACS Appl. Mater. Interfaces 17, 16616–16631. 10.1021/acsami.4c20010 40048359 PMC11931483

[B76] Liu Y. Li P. Jiang T. Li Y. Wang Y. Cheng Z. (2023). Epidermal growth factor receptor in asthma: a promising therapeutic target? Respir. Med. 207, 107117. 10.1016/j.rmed.2023.107117 36626942

[B77] Lula I. Denadai Â. L. Resende J. M. De Sousa F. B. De Lima G. F. Pilo-Veloso D. (2007). Study of angiotensin-(1–7) vasoactive peptide and its β-cyclodextrin inclusion complexes: complete sequence-specific NMR assignments and structural studies. Peptides 28, 2199–2210. 10.1016/j.peptides.2007.08.011 17904691

[B78] Magalhães G. S. Rodrigues‐Machado M. G. Motta‐Santos D. Silva A. R. Caliari M. V. Prata L. O. (2015). A ngiotensin‐(1‐7) attenuates airway remodelling and hyperresponsiveness in a model of chronic allergic lung inflammation. Br. J Pharmacol. 172, 2330–2342. 10.1111/bph.13057 25559763 PMC4403097

[B79] Magalhães G. S. Rodrigues-Machado M. G. Motta-Santos D. Alenina N. Bader M. Santos R. A. (2016). Chronic allergic pulmonary inflammation is aggravated in angiotensin-(1-7) mas receptor knockout mice. Am. J. Physiol. Lung Cell Mol. Physiol. 311, L1141–L1148. 10.1152/ajplung.00029.2016 27815255

[B80] Magalhães G. S. Barroso L. C. Reis A. C. Rodrigues-Machado M. G. Gregório J. F. Motta-Santos D. (2018). Angiotensin-(1–7) promotes resolution of eosinophilic inflammation in an experimental model of asthma. Front. Immunol. 9, 58. 10.3389/fimmu.2018.00058 29434591 PMC5797293

[B81] Magalhães G. S. Gregório J. F. Ramos K. E. Cançado-Ribeiro A. T. P. Baroni I. F. Barcelos L. S. (2020). Treatment with inhaled formulation of angiotensin-(1-7) reverses inflammation and pulmonary remodeling in a model of chronic asthma. Immunobiology 225, 151957. 10.1016/j.imbio.2020.151957 32517880

[B82] Magalhães G. S. Gregório J. F. Cançado Ribeiro A. T. P. Baroni I. F. Vasconcellos A. V. D. O. Nakashima G. P. (2021). Oral formulation of Angiotensin-(1-7) promotes therapeutic actions in a model of eosinophilic and neutrophilic asthma. Front. Pharmacol. 12, 557962. 10.3389/fphar.2021.557962 33762930 PMC7982577

[B83] Magalhães G. S. Gregorio J. F. Beltrami V. A. Felix F. B. Oliveira-Campos L. Bonilha C. S. (2024). A single dose of angiotensin-(1–7) resolves eosinophilic inflammation and protects the lungs from a secondary inflammatory challenge. Inflamm. Res. 73, 1019–1031. 10.1007/s00011-024-01880-x 38656426

[B84] Magalhães G. S. Villacampa A. Rodrigues-Machado M. G. Campagnole-Santos M. J. Souza Santos R. A. Sánchez-Ferrer C. F. (2025). Oral Angiotensin-(1-7) formulation after established elastase-induced emphysema suppresses inflammation and restores lung architecture. Front. Pharmacol. 16, 1540475. 10.3389/fphar.2025.1540475 40606618 PMC12213791

[B85] Malhotra A. Hepokoski M. McCowen K. C. Y-J Shyy J. (2020). ACE2, metformin, and COVID-19. iScience 23, 101425. 10.1016/j.isci.2020.101425 32818905 PMC7452173

[B86] Marques F. D. Ferreira A. J. Sinisterra R. D. M. Jacoby B. A. Sousa F. B. Caliari M. V. (2011). An oral formulation of Angiotensin-(1-7) produces cardioprotective effects in infarcted and isoproterenol-treated rats. Hypertension 57, 477–483. 10.1161/HYPERTENSIONAHA.110.167346 21282558

[B87] Martins A. L. V. Annoni F. Da Silva F. A. Bolais-Ramos L. De Oliveira G. C. Ribeiro R. C. (2024). Angiotensin-(1–7) infusion in COVID-19 patients admitted to the ICU: a seamless phase 1–2 randomized clinical trial. Ann. Intensive Care 14, 139. 10.1186/s13613-024-01369-0 39231898 PMC11374945

[B88] McCollum L. T. Gallagher P. E. Ann Tallant E. (2012). Angiotensin-(1–7) attenuates angiotensin II-induced cardiac remodeling associated with upregulation of dual-specificity phosphatase 1. Am. J. Physiology-Heart Circulatory Physiology 302, H801–H810. 10.1152/ajpheart.00908.2011 22140049 PMC3353789

[B89] Mendes S. Guimarães L. C. Costa P. A. C. Fernandez C. C. Figueiredo M. M. Teixeira M. M. (2024). Intranasal liposomal angiotensin-(1-7) administration reduces inflammation and viral load in the lungs during SARS-CoV-2 infection in K18-hACE2 transgenic mice. Antimicrob. Agents Chemother. 68, e00835-24. 10.1128/aac.00835-24 39470198 PMC11619396

[B90] Meng Y. Yu C.-H. Li W. Li T. Luo W. Huang S. (2014). Angiotensin-converting enzyme 2/Angiotensin-(1-7)/Mas axis protects against lung fibrosis by inhibiting the MAPK/NF-κB pathway. Am. J. Respir. Cell Mol. Biol. 50, 723–736. 10.1165/rcmb.2012-0451OC 24168260

[B91] Meng Y. Li T. Zhou G. Chen Y. Yu C.-H. Pang M.-X. (2015). The angiotensin-converting enzyme 2/Angiotensin (1–7)/Mas axis protects against lung fibroblast migration and lung fibrosis by inhibiting the NOX4-Derived ROS-mediated RhoA/Rho kinase pathway. Antioxidants and Redox Signal. 22, 241–258. 10.1089/ars.2013.5818 25089563 PMC4283064

[B92] Mińczuk K. Schlicker E. Krzyżewska A. Malinowska B. (2025). Angiotensin 1-7 injected into the rat paraventricular nucleus of hypothalamus increases blood pressure and heart rate *via* various receptors. Neuropharmacology 266, 110279. 10.1016/j.neuropharm.2024.110279 39732324

[B93] Mirza K. Landoski K. Thakar D. Heir-Singh J. Jackson T. Kassab C. (2020). Sugammadex-associated hypotension, bradycardia, asystole, and death. Case Rep. Anesthesiol. 2020, 1–2. 10.1155/2020/8767195 32566314 PMC7303763

[B94] Mordwinkin N. M. Russell J. R. Burke A. S. Dizerega G. S. Louie S. G. Rodgers K. E. (2012). Toxicological and toxicokinetic analysis of angiotensin (1–7) in two species. J. Pharm. Sci. 101, 373–380. 10.1002/jps.22730 21858825 PMC3619381

[B95] Mori J. Patel V. B. Abo Alrob O. Basu R. Altamimi T. DesAulniers J. (2014). Angiotensin 1–7 ameliorates diabetic cardiomyopathy and diastolic dysfunction in *db/db* mice by reducing lipotoxicity and inflammation. Circ. Heart Fail. 7, 327–339. 10.1161/CIRCHEARTFAILURE.113.000672 24389129

[B96] Muthalif M. M. Benter I. F. Uddin M. R. Malik K. U. (1996). Calcium/calmodulin-Dependent protein kinase IIα mediates activation of mitogen-activated protein kinase and cytosolic phospholipase A2 in norepinephrine-induced arachidonic acid release in rabbit aortic smooth muscle cells. J. Biol. Chem. 271, 30149–30157. 10.1074/jbc.271.47.30149 8939965

[B97] Muthalif M. M. Benter I. F. Uddin M. R. Harper J. L. Malik K. U. (1998). Signal transduction mechanisms involved in angiotensin-(1-7)-stimulated arachidonic acid release and prostanoid synthesis in rabbit aortic smooth muscle cells. J. Pharmacol. Exp. Ther. 284, 388–398. 9435202

[B98] Nabe T. (2020). Steroid-resistant asthma and neutrophils. Biol. and Pharm. Bull. 43, 31–35. 10.1248/bpb.b19-00095 31902928

[B99] Nell L. M. Hendriks R. W. Wijsenbeek M. S. Corneth O. B. J. Koudstaal T. (2026). Immunological mechanisms and therapeutic approaches in pulmonary fibrosis. Eur. Respir. Rev. 35 (180), 250227. 10.1183/16000617.0227-2025 41951242 PMC13058739

[B100] Oh J. Kim S. Kim M. S. Abate Y. H. Abd ElHafeez S. Abdelkader A. (2025). Global, regional, and national burden of asthma and atopic dermatitis, 1990–2021, and projections to 2050: a systematic analysis of the global burden of disease study 2021. Lancet Respir. Med. 13, 425–446. 10.1016/S2213-2600(25)00003-7 40147466

[B101] Okada H. (2011). Drug discovery by formulation design and innovative drug delivery systems (DDS). YAKUGAKU ZASSHI 131, 1271–1287. 10.1248/yakushi.131.1271 21881300

[B102] Onal H. Ergun N. U. Arslan B. Topuz S. Semerci S. Y. Ugurel O. M. (2022). Angiotensin (1−7) peptide replacement therapy with plasma transfusion in COVID-19. Transfus. Apher. Sci. 61, 103418. 10.1016/j.transci.2022.103418 35305923 PMC8917875

[B103] Pan M. Zheng Z. Chen Y. Sun N. Zheng B. Yang Q. (2018). Angiotensin-(1-7) attenuated cigarette smoking–related pulmonary fibrosis *via* improving the impaired autophagy caused by nicotinamide adenine dinucleotide phosphate reduced oxidase 4–Dependent reactive oxygen species. Am. J. Respir. Cell Mol. Biol. 59, 306–319. 10.1165/rcmb.2017-0284OC 29652517

[B104] Petty W. J. Miller A. A. McCoy T. P. Gallagher P. E. Tallant E. A. Torti F. M. (2009). Phase I and pharmacokinetic study of Angiotensin-(1-7), an endogenous antiangiogenic hormone. Clin. Cancer Res. 15, 7398–7404. 10.1158/1078-0432.CCR-09-1957 19920106 PMC3703919

[B105] Pham H. Schwartz B. M. Delmore J. E. Reed E. Cruickshank S. Drummond L. (2013). Pharmacodynamic stimulation of thrombogenesis by angiotensin (1–7) in recurrent ovarian cancer patients receiving gemcitabine and platinum-based chemotherapy. Cancer Chemother. Pharmacol. 71, 965–972. 10.1007/s00280-013-2089-x 23370663

[B106] Plovsing R. R. Wamberg C. Sandgaard N. C. F. Simonsen J. A. Holstein-Rathlou N.-H. Høilund-Carlsen P. F. (2003). Effects of truncated angiotensins in humans after double blockade of the renin system. Am. J. Physiology-Regulatory, Integr. Comp. Physiology 285, R981–R991. 10.1152/ajpregu.00263.2003 12869368

[B107] Prasad A. Quyyumi A. A. (2004). Renin-angiotensin system and angiotensin receptor blockers in the metabolic syndrome. Circulation 110, 1507–1512. 10.1161/01.CIR.0000141736.76561.78 15364819

[B108] Qi P. Bu R. Zhang H. Yin J. Chen J. Zhang A. (2019). Goserelin acetate loaded poloxamer hydrogel in PLGA microspheres: core–shell Di-Depot intramuscular sustained release delivery system. Mol. Pharm. 16, 3502–3513. 10.1021/acs.molpharmaceut.9b00344 31251642

[B109] Qiao Q. Liu X. Yang T. Cui K. Kong L. Yang C. (2021). Nanomedicine for acute respiratory distress syndrome: the latest application, targeting strategy, and rational design. Acta Pharm. Sin. B 11, 3060–3091. 10.1016/j.apsb.2021.04.023 33977080 PMC8102084

[B110] Rago F. Melo E. M. Kraemer L. Galvão I. Cassali G. D. Santos R. A. S. (2019). Effect of preventive or therapeutic treatment with angiotensin 1–7 in a model of bleomycin-induced lung fibrosis in mice. J. Leukoc. Biol. 106, 677–686. 10.1002/JLB.MA1218-490RR 31256436

[B111] Rendell M. (2014). Technosphere inhaled insulin (afrezza). Drugs Today 50, 813–827. 10.1358/dot.2014.50.12.2233894 25588086

[B112] Robbins A. J. Che Bakri N. A. Toke‐Bjolgerud E. Edwards A. Vikraman A. Michalsky C. (2023). The effect of TRV027 on coagulation in COVID‐19: a pilot randomized, placebo‐controlled trial. Brit J. Clin. Pharma 89, 1495–1501. 10.1111/bcp.15618 PMC10952550 36437688

[B113] Rodgers K. E. Oliver J. diZerega G. S. (2006). Phase I/II dose escalation study of angiotensin 1-7 [A(1-7)] administered before and after chemotherapy in patients with newly diagnosed breast cancer. Cancer Chemother. Pharmacol. 57, 559–568. 10.1007/s00280-005-0078-4 16096787

[B114] Rodrigues‐Machado M. G. Magalhães G. S. Cardoso J. A. Kangussu L. M. Murari A. Caliari M. V. (2013). AVE 0991, a non‐peptide mimic of angiotensin‐(1–7) effects, attenuates pulmonary remodelling in a model of chronic asthma. Br. J Pharmacol. 170, 835–846. 10.1111/bph.12318 23889691 PMC3799597

[B115] Rosalia M. Chiesa E. Tottoli E. M. Dorati R. Genta I. Conti B. (2022). Tobramycin nanoantibiotics and their advantages: a minireview. IJMS 23, 14080. 10.3390/ijms232214080 36430555 PMC9692674

[B116] Santos R. A. S. E Silva A. C. S. Maric C. Silva D. M. R. Machado R. P. De Buhr I. (2003). Angiotensin-(1–7) is an endogenous ligand for the G protein-coupled receptor mas. Proc. Natl. Acad. Sci. U.S.A. 100, 8258–8263. 10.1073/pnas.1432869100 12829792 PMC166216

[B117] Santos R. A. S. Sampaio W. O. Alzamora A. C. Motta-Santos D. Alenina N. Bader M. (2018). The ACE2/Angiotensin-(1–7)/MAS axis of the renin-angiotensin system: focus on Angiotensin-(1–7). Physiol. Rev. 98, 505–553. 10.1152/physrev.00023.2016 29351514 PMC7203574

[B118] Sasaki S. Higashi Y. Nakagawa K. Matsuura H. Kajiyama G. Oshima T. (2001). Effects of Angiotensin-(1-7) on forearm circulation in normotensive subjects and patients with essential hypertension. Hypertension 38, 90–94. 10.1161/01.HYP.38.1.90 11463766

[B119] Savin I. A. Zenkova M. A. Sen’kova A. V. (2023). Bronchial asthma, airway remodeling and lung fibrosis as successive steps of one process. IJMS 24, 16042. 10.3390/ijms242216042 38003234 PMC10671561

[B120] Schwacke J. H. Spainhour J. C. G. Ierardi J. L. Chaves J. M. Arthur J. M. Janech M. G. (2013). Network modeling reveals steps in angiotensin peptide processing. Hypertension 61, 690–700. 10.1161/HYPERTENSIONAHA.111.00318 23283355 PMC5001166

[B121] Sheng M. Li Q. Huang W. Yu D. Pan H. Qian K. (2024). Ang-(1–7)/Mas axis ameliorates bleomycin-induced pulmonary fibrosis in mice *via* restoration of Nox4-Nrf2 redox homeostasis. Eur. J. Pharmacol. 962, 176233. 10.1016/j.ejphar.2023.176233 38043775

[B122] Shenoy V. Ferreira A. J. Qi Y. Fraga-Silva R. A. Díez-Freire C. Dooies A. (2010). The angiotensin-converting enzyme 2/Angiogenesis-(1–7)/Mas axis confers cardiopulmonary protection against lung fibrosis and pulmonary hypertension. Am. J. Respir. Crit. Care Med. 182, 1065–1072. 10.1164/rccm.200912-1840OC 20581171 PMC2970847

[B123] Sheth V. Wang L. Bhattacharya R. Mukherjee P. Wilhelm S. (2021). Strategies for delivering nanoparticles across tumor blood vessels. Adv. Funct. Mater. 31, 2007363. 10.1002/adfm.202007363 37197212 PMC10187772

[B124] Shraim B. A. Moursi M. O. Benter I. F. Habib A. M. Akhtar S. (2021). The role of epidermal growth factor receptor family of receptor tyrosine kinases in mediating diabetes-induced cardiovascular complications. Front. Pharmacol. 12, 701390. 10.3389/fphar.2021.701390 34408653 PMC8365470

[B125] Silva De Moura S. De Assis Dias Martins-Júnior F. Cruz De Oliveira E. Coelho D. B. Boari D. Lima-Silva A. E. (2024). Effects of oral HPΒCD-angiotensin-(1-7) supplementation on recreational Mountain bike athletes: a crossover study. Physician Sportsmed. 52, 65–76. 10.1080/00913847.2023.2175587 36752064

[B126] Singh K. D. Karnik S. S. (2024). Implications of β-Arrestin biased signaling by angiotensin II type 1 receptor for cardiovascular drug discovery and therapeutics. Cell. Signal. 124, 111410. 10.1016/j.cellsig.2024.111410 39270918

[B127] Sousa De Almeida M. Susnik E. Drasler B. Taladriz-Blanco P. Petri-Fink A. Rothen-Rutishauser B. (2021). Understanding nanoparticle endocytosis to improve targeting strategies in nanomedicine. Chem. Soc. Rev. 50, 5397–5434. 10.1039/D0CS01127D 33666625 PMC8111542

[B128] Spikes L. A. Bajwa A. A. Burger C. D. Desai S. V. Eggert M. S. El‐Kersh K. A. (2022). BREEZE: open‐label clinical study to evaluate the safety and tolerability of treprostinil inhalation powder as tyvaso DPI^TM^ in patients with pulmonary arterial hypertension. Pulm. Circ 12, e12063. 10.1002/pul2.12063 35514770 PMC9063953

[B129] Su C. Li C. Hu X. Wang J. Liu L. Zhang X. (2024). Association between ACE2 and lung diseases. IDR 17, 1771–1780. 10.2147/IDR.S445180 PMC11088384 38736435

[B130] Subbiah R. Hwang M. P. Van S. Y. Do S. H. Park H. Lee K. (2015). Osteogenic/angiogenic dual growth factor delivery microcapsules for regeneration of vascularized bone tissue. Adv. Healthc. Mater. 4, 1982–1992. 10.1002/adhm.201500341 26138344

[B131] Sun N.-N. Yu C.-H. Pan M.-X. Zhang Y. Zheng B.-J. Yang Q.-J. (2017). Mir-21 mediates the inhibitory effect of ang (1–7) on AngII-induced NLRP3 inflammasome activation by targeting Spry1 in lung fibroblasts. Sci. Rep. 7, 14369. 10.1038/s41598-017-13305-3 29084974 PMC5662719

[B132] Supé S. Kohse F. Gembardt F. Kuebler W. M. Walther T. (2016). Therapeutic time window for angiotensin‐(1–7) in acute lung injury. Br. J Pharmacol. 173, 1618–1628. 10.1111/bph.13462 26895462 PMC4842919

[B133] Tawengi M. Al-Dali Y. Tawengi A. Benter I. F. Akhtar S. (2024). Targeting the epidermal growth factor receptor (EGFR/ErbB) for the potential treatment of renal pathologies. Front. Pharmacol. 15, 1394997. 10.3389/fphar.2024.1394997 39234105 PMC11373609

[B134] Tipnis S. R. Hooper N. M. Hyde R. Karran E. Christie G. Turner A. J. (2000). A human homolog of angiotensin-converting enzyme. Cloning and functional expression as a captopril-insensitive carboxypeptidase. J. Biol. Chem. 275, 33238–33243. 10.1074/jbc.M002615200 10924499

[B135] Tirupula K. C. Desnoyer R. Speth R. C. Karnik S. S. (2014). Atypical signaling and functional desensitization response of MAS receptor to peptide ligands. PLoS One 9, e103520. 10.1371/journal.pone.0103520 25068582 PMC4113456

[B136] Trachalaki A. Lindahl A. L. Petrarulo S. Margaritopoulos G. A. (2025). “regression to the truth”: lessons learned from negative IPF trials. Breathe 21, 240260. 10.1183/20734735.0260-2024 40255293 PMC12004256

[B137] Valentini A. Heilmann R. M. Kühne A. Biagini L. De Bellis D. Rossi G. (2025). The renin–angiotensin–aldosterone system (RAAS): beyond cardiovascular regulation. Veterinary Sci. 12, 777. 10.3390/vetsci12080777 PMC12390185 40872727

[B138] Van Twist D. J. L. Houben A. J. H. M. De Haan M. W. Mostard G. J. M. Kroon A. A. De Leeuw P. W. (2013). Angiotensin-(1–7)–Induced renal vasodilation in hypertensive humans is attenuated by low sodium intake and angiotensin II Co-Infusion. Hypertension 62, 789–793. 10.1161/HYPERTENSIONAHA.113.01814 23918750

[B139] Vasileiadis I. E. Goudis C. A. Giannakopoulou P. T. Liu T. (2018). Angiotensin converting enzyme inhibitors and angiotensin receptor blockers: a promising medication for chronic obstructive pulmonary disease? COPD J. Chronic Obstr. Pulm. Dis. 15, 148–156. 10.1080/15412555.2018.1432034 29521545

[B140] Wagener G. Goldklang M. P. Gerber A. Elisman K. Eiseman K. A. Fonseca L. D. (2022). A randomized, placebo-controlled, double-blinded pilot study of angiotensin 1–7 (TXA-127) for the treatment of severe COVID-19. Crit. Care 26, 229. 10.1186/s13054-022-04096-9 35902867 PMC9332096

[B141] Wen Y. Nie J. Qin X. Li Z. (2025). Functional metabolomics revealed pyroglutamic acid May play a key role in idiopathic pulmonary fibrosis. J. Pharm. Biomed. Analysis 264, 116967. 10.1016/j.jpba.2025.116967 40398246

[B142] Wester A. Devocelle M. Tallant E. A. Chappell M. C. Gallagher P. E. Paradisi F. (2017). Stabilization of Angiotensin-(1–7) by key substitution with a cyclic non-natural amino acid. Amino Acids 49, 1733–1742. 10.1007/s00726-017-2471-9 28744580 PMC9239115

[B143] Xu J. Yu Z. Liu X. (2023). Angiotensin-(1–7) suppresses airway inflammation and airway remodeling *via* inhibiting ATG5 in allergic asthma. BMC Pulm. Med. 23, 422. 10.1186/s12890-023-02719-7 37919667 PMC10623740

[B144] Yousif M. H. M. Dhaunsi G. S. Makki B. M. Qabazard B. A. Akhtar S. Benter I. F. (2012). Characterization of Angiotensin-(1–7) effects on the cardiovascular system in an experimental model of Type-1 diabetes. Pharmacol. Res. 66, 269–275. 10.1016/j.phrs.2012.05.001 22580236

[B145] Yousif M. H. M. Makki B. El-Hashim A. Z. Akhtar S. Benter I. F. (2014). Chronic treatment with Ang-(1-7) reverses abnormal reactivity in the corpus cavernosum and normalizes diabetes-induced changes in the protein levels of ACE, ACE2, ROCK1, ROCK2 and omega-hydroxylase in a rat model of type 1 diabetes. J. Diabetes Res. 2014, 1–10. 10.1155/2014/142154 25309930 PMC4182022

[B146] Yuan D. Li Q. Zhang Q. Zhou F. Zhao Q. Zhao M. (2024). Enhanced curcumin transportation across epithelial barrier by mucus-permeable soy protein nanoparticles-mediated dual transcytosis pathways. Food Chem. 437, 137771. 10.1016/j.foodchem.2023.137771 37897825

[B147] Zambelli V. Bellani G. Borsa R. Pozzi F. Grassi A. Scanziani M. (2015). Angiotensin-(1-7) improves oxygenation, while reducing cellular infiltrate and fibrosis in experimental acute respiratory distress syndrome. ICMx 3, 8. 10.1186/s40635-015-0044-3 26215809 PMC4512981

[B148] Zhang Y. Li Y. Shi C. Fu X. Zhao L. Song Y. (2018). Angiotensin‐(1‐7)‐mediated Mas1 receptor/NF‐κB‐p65 signaling is involved in a cigarette smoke‐induced chronic obstructive pulmonary disease mouse model. Environ. Toxicol. 33, 5–15. 10.1002/tox.22454 28960804

[B149] Zhang B. Xu H. Zhang Y. Yi X. Zhang G. Zhang X. (2019). Targeting the RAS axis alleviates silicotic fibrosis and ang II-induced myofibroblast differentiation *via* inhibition of the hedgehog signaling pathway. Toxicol. Lett. 313, 30–41. 10.1016/j.toxlet.2019.05.023 31181250

[B150] Zhang R. Zhang M. Chen L. Jiang L. Zou C. Li N. (2024). Dual-phase SilMA hydrogel: a dynamic scaffold for sequential drug release and enhanced spinal cord repair *via* neural differentiation and immunomodulation. Front. Bioeng. Biotechnol. 12, 1501488. 10.3389/fbioe.2024.1501488 39640066 PMC11617199

[B151] Zhou J. P. Wang Y. Li S. Q. Zhang J. Q. Lin Y. N. Sun X. W. (2023). Exogenous Ang-(1-7) inhibits autophagy *via* HIF-1α/THBS1/BECN1 axis to alleviate chronic intermittent hypoxia-enhanced airway remodelling of asthma. Cell Death Discov. 9, 366. 10.1038/s41420-023-01662-0 37783703 PMC10545676

[B152] Zimmerman D. L. Zimpelmann J. Xiao F. Gutsol A. Touyz R. Burns K. D. (2015). The effect of Angiotensin-(1-7) in mouse unilateral ureteral obstruction. Am. J. Pathology 185, 729–740. 10.1016/j.ajpath.2014.11.013 25625676

