# Supplementary material for: Angiotensin-(1-7) in asthma and pulmonary fibrosis: mechanisms of action, clinical safety, and nano-delivery strategies
Source: Front Pharmacol. 2026 Aug 3;17:1789833. doi: 10.3389/fphar.2026.1789833 (PMC13477901; doi:10.3389/fphar.2026.1789833)
Supplement: Supplementary file 1 [file Table1.docx]

Khanafer Y, Dalol A, El-Hashim AZ, Benter IF and Akhtar S (2026) Angiotensin-(1-7) in asthma and pulmonary fibrosis:

mechanisms of action, clinical safety, and nano-delivery strategies. Front. Pharmacol. 17:1789833.

**Abbreviations (Supplementary table)**

*The following abbreviations apply to Tables 1 to 4.*

| **α-SMA:** alpha-smooth muscle actin | **EPO:** eosinophil peroxidase | **NEFA:** non-esterified fatty acid |
| --- | --- | --- |
| **ACE / ACE2:** angiotensin-converting enzyme / enzyme 2 | **ERK1/2:** extracellular signal-regulated kinase 1/2 | **NF-κB:** nuclear factor kappa B |
| **Ac-SDKP:** N-acetyl-seryl-aspartyl-lysyl-proline | **FBF:** forearm blood flow | **NLRP3:** NLR family pyrin domain containing 3 |
| **AE / SAE:** adverse event / serious adverse event | **FeNO:** fractional exhaled nitric oxide | **NO:** nitric oxide |
| **AHR:** airway hyperresponsiveness | **FEV₁:** forced expiratory volume in 1 s | **NOX4:** NADPH oxidase 4 |
| **AKI:** acute kidney injury | **FPF / MMAD:** fine particle fraction / mass median aerodynamic diameter | **NQO1:** NAD(P)H quinone dehydrogenase 1 |
| **ALI / ARDS:** acute lung injury / acute respiratory distress syndrome | **GATA3:** GATA-binding protein 3 | **NR:** not reported |
| **Ang II:** angiotensin II | **GFR:** glomerular filtration rate | **Nrf2:** nuclear factor erythroid 2-related factor 2 |
| **Ang-(1-7):** angiotensin-(1-7) | **GLI:** glioma-associated oncogene homolog | **OBD:** optimal biological dose |
| **AT1R:** angiotensin II type 1 receptor | **GM-CSF:** granulocyte-macrophage colony-stimulating factor | **OSA:** obstructive sleep apnea |
| **ATG5:** autophagy-related protein 5 | **H₂O₂:** hydrogen peroxide | **OVA:** ovalbumin |
| **A779:** D-Ala⁷-Ang-(1-7); Mas receptor antagonist | **HIF-1α:** hypoxia-inducible factor 1-alpha | **p- (prefix):** phosphorylated |
| **AVE 0991:** synthetic non-peptide Mas receptor agonist | **HO-1:** heme oxygenase-1 | **PF:** pulmonary fibrosis |
| **BAL / BALF:** bronchoalveolar lavage / lavage fluid | **HPβCD:** hydroxypropyl β-cyclodextrin | **PH:** pulmonary hypertension |
| **BLM:** bleomycin | **HPBMC:** human peripheral blood mononuclear cells | **PK:** pharmacokinetics |
| **BP / HR:** blood pressure / heart rate | **IF:** immunofluorescence | **PNA5:** glycosylated Ang-(1-7) Mas receptor agonist |
| **CCL:** C-C motif chemokine ligand | **IgE:** immunoglobulin E | **PRA:** plasma renin activity |
| **CIH:** chronic intermittent hypoxia | **IL:** interleukin | **PTC:** Patched |
| **CK:** creatine kinase | **IP / IT:** intraperitoneal / intratracheal | **RAAS / RAS:** renin-angiotensin(-aldosterone) system |
| **Cmax:** maximum plasma concentration | **IV / SC:** intravenous / subcutaneous | **ROS:** reactive oxygen species |
| **COPD:** chronic obstructive pulmonary disease | **IκB-α:** inhibitor of kappa B alpha | **RV / RVSP:** right ventricle / right ventricular systolic pressure |
| **CSE:** cigarette smoke extract | **JNK:** c-Jun N-terminal kinase | **SHH / HH:** Sonic hedgehog / Hedgehog |
| **CTGF:** connective tissue growth factor | **Keap1:** Kelch-like ECH-associated protein 1 | **SMO:** Smoothened |
| **CTRL:** control | **LC3 / LC3B:** microtubule-associated protein 1 light chain 3 (beta) | **Spry1:** Sprouty1 |
| **DLT:** dose-limiting toxicity | **LPS:** lipopolysaccharide | **Src:** Src kinase |
| **DRC:** dose-response curve | **MAPK:** mitogen-activated protein kinase | **TGF-β1:** transforming growth factor beta 1 |
| **DVT:** deep vein thrombosis | **Mas / Mas1:** Mas receptor | **Tg:** glass-transition temperature |
| **ECM:** extracellular matrix | **MCT:** monocrotaline | **THBS1:** thrombospondin 1 |
| **ECOG:** Eastern Cooperative Oncology Group | **MDA:** malondialdehyde | **TNF-α:** tumour necrosis factor alpha |
| **EE / PDI:** encapsulation efficiency / polydispersity index | **meprin-α:** meprin alpha (Ac-SDKP synthetase) | **Treg:** regulatory T cell |
| **EGFR:** epidermal growth factor receptor | **miR-21:** microRNA-21 | **TRV027 / TXA-127:** Ang-(1-7) receptor agonist / formulation |
| **EMG:** electromyography | **MMP:** matrix metalloproteinase | **VEGF:** vascular endothelial growth factor |
| **EMT:** epithelial-mesenchymal transition | **MPO:** myeloperoxidase | **WT:** wild-type |
